# Hepatitis B virus pathogenesis relevant immunosignals uncovering amino acids utilization related risk factors guide artificial intelligence-based precision medicine

**DOI:** 10.3389/fphar.2022.1079566

**Published:** 2022-12-09

**Authors:** Jun Huang, Chunbei Zhao, Xinhe Zhang, Qiaohui Zhao, Yanting Zhang, Liping Chen, Guifu Dai

**Affiliations:** ^1^ School of Life Sciences, Zhengzhou University, Zhengzhou, Henan, China; ^2^ Key Laboratory of Gastroenterology and Hepatology, State Key Laboratory for Oncogenes and Related Genes, Department of Gastroenterology and Hepatology, Ministry of Health, Shanghai Institute of Digestive Disease, Renji Hospital, School of Medicine, Shanghai Jiaotong University, Shanghai, China; ^3^ Shanghai Public Health Clinical Center, Fudan University, Shanghai, China

**Keywords:** hepatitis B virus, hepatocellular carcinoma, tumor microenvironment (TME), artificial inteligence-AI, anti-tumor drug, prognosis, amino acids utilization

## Abstract

**Background:** Although immune microenvironment-related chemokines, extracellular matrix (ECM), and intrahepatic immune cells are reported to be highly involved in hepatitis B virus (HBV)-related diseases, their roles in diagnosis, prognosis, and drug sensitivity evaluation remain unclear. Here, we aimed to study their clinical use to provide a basis for precision medicine in hepatocellular carcinoma (HCC) *via* the amalgamation of artificial intelligence.

**Methods:** High-throughput liver transcriptomes from Gene Expression Omnibus (GEO), NODE (https://www.bio.sino.org/node), the Cancer Genome Atlas (TCGA), and our in-house hepatocellular carcinoma patients were collected in this study. Core immunosignals that participated in the entire diseases course of hepatitis B were explored using the “Gene set variation analysis” R package. Using ROC curve analysis, the impact of core immunosignals and amino acid utilization related gene on hepatocellular carcinoma patient’s clinical outcome were calculated. The utility of core immunosignals as a classifier for hepatocellular carcinoma tumor tissue was evaluated using explainable machine-learning methods. A novel deep residual neural network model based on immunosignals was constructed for the long-term overall survival (LS) analysis. *In vivo* drug sensitivity was calculated by the “oncoPredict” R package.

**Results:** We identified nine genes comprising chemokines and ECM related to hepatitis B virus-induced inflammation and fibrosis as CLST signals. Moreover, CLST was co-enriched with activated CD4+ T cells bearing harmful factors (aCD4) during all stages of hepatitis B virus pathogenesis, which was also verified by our hepatocellular carcinoma data. Unexpectedly, we found that hepatitis B virus-hepatocellular carcinoma patients in the CLST^high^aCD4^high^ subgroup had the shortest overall survival (OS) and were characterized by a risk gene signature associated with amino acids utilization. Importantly, characteristic genes specific to CLST/aCD4 showed promising clinical relevance in identifying patients with early-stage hepatocellular carcinoma *via* explainable machine learning. In addition, the 5-year long-term overall survival of hepatocellular carcinoma patients can be effectively classified by CLST/aCD4 based GeneSet-ResNet model. Subgroups defined by CLST and aCD4 were significantly involved in the sensitivity of hepatitis B virus-hepatocellular carcinoma patients to chemotherapy treatments.

**Conclusion:** CLST and aCD4 are hepatitis B virus pathogenesis-relevant immunosignals that are highly involved in hepatitis B virus-induced inflammation, fibrosis, and hepatocellular carcinoma. Gene set variation analysis derived immunogenomic signatures enabled efficient diagnostic and prognostic model construction. The clinical application of CLST and aCD4 as indicators would be beneficial for the precision management of hepatocellular carcinoma.

## Introduction

Chronic hepatitis B virus (HBV) infection remains a major health concern worldwide (Kramvis et al., 2022). First-line anti-HBV drugs approved by FDA including PEG IFN-α and nucleoside (acid) analogs (NAs) are not yet effective in achieving functional cure referring to hepatitis B surface antigen (HBsAg) and covalently closed circular DNA (cccDNA) elimination (Levrero et al., 2018; Fanning et al., 2019; Yang et al., 2019; Tout et al., 2020). Over 200 million people are afflicted with chronic hepatitis B (CHB) and are at a high risk of developing liver fibrosis (LF), liver cirrhosis, and hepatocellular carcinoma (HCC) (Wangensteen and Chang, 2021). HBV-related diseases cause heavy economic pressure and psychological burden to many families, especially in the Asia-Pacific region, where HBV is highly prevalent (Wang et al., 2017; Wong et al., 2019; Howell et al., 2020; Sarin et al., 2020). Considerable evidence suggests that chemokines, the extracellular matrix (ECM), parenchymal hepatic cells, tissue-resident lymphocytes, and extrahepatic immune cells in the liver microenvironment are associated with HBV-related diseases progression (Yuen et al., 2018). CXCR3-related chemokines (CXCL9 and CXCL10), directly produced by hepatocytes or liver sinusoidal endothelial cells at the early stage of HBV infection, can result in intrahepatic lymphocyte infiltration (Rehermann, 2013). SPP1(the CD44 ligand) derived from activated hepatic stellate cells (HSC) serves as a stimulator for KLRG1+ NK cells that can mediate liver scarring limitation in CHB pathogenesis (Wijaya et al., 2019) and has predictive value in the prognosis of HCC (Shang et al., 2012; da Costa et al., 2015). SOX9, which can be directly induced in HBV-infected human hepatoma cells (Yang et al., 2020) has been identified as a risk factor for cirrhosis and HCC (Chen et al., 2021; Damrauer et al., 2021). However, these previous studies are performed just through flow cytometry (FCM), immune fluorescence (IF), and immunohistochemistry (IHC) with limited subpopulations of liver-infiltrating lymphocytes (LILs) and a small samples size; the orchestra of multiple chemokines and ECM related genes with a variety of LILs during HBV pathogenesis are not globally indicated.

The core mechanism underlying amino acid metabolic adaptations in cancer cells to grow in a nutrient-deficient tumor microenvironment (TME) was recently reported, and LYSET (TMEM251) and other amino acid utilization-associated genes (ATF4, TSC2, VPS18, RAB7A, SLC7A5, SLC3A2, TGFBRAP1, GNPTAB, and GCN2) have been primarily screened out mainly through CRISPR-Cas9 based high-throughput method (Pechincha et al., 2022). Although these key players essential for tumor cell proliferation in harsh TME conditions and LYSET invovled in lysosomal biogenesis have been uncovered in the latest studies (Pechincha et al., 2022; Richards et al., 2022), their impact on pan-cancer clinical outcomes remains unknown. The metabolic status of amino acids in HCC patients with different immune subtypes according to HBV pathogenesis-relevant immunosignals is worthy of further study.

Currently, precise diagnosis and prognosis of HBV-related liver diseases have attracted much attention (Petrizzo et al., 2018; Zheng et al., 2020). The main obstacle to artificial intelligence (AI) models’ establishment in genome medicine is that neither gene microarray nor RNA-seq data are suitable for direct learning (Oh et al., 2021). Although several AI models based on these high-dimensional biological data have been constructed to detect liver cancer at an early stage and assess the prognosis (Long et al., 2019; Tao et al., 2020; Christou and Tsoulfas, 2021; Liu W. et al., 2021; Liu X. et al., 2021), the input data used in these models are relatively complex and not easy to follow. Until now, the optimal model with a promising predictive value for clinical utilization has been far from reaching a general consensus (Le Berre et al., 2020; Christou and Tsoulfas, 2021; Liu X. et al., 2021; Oh et al., 2021; Wang et al., 2022). “Gene set variation analysis (GSVA)” R package (GSVA, for short) (Aran et al., 2017; Charoentong et al., 2017), CIBERSORT (Newman et al., 2015), MCP-counter and TIMER were primarily developed and used for novel immune cell subtype identification and concentration evaluation using tissue transcriptome data (Aran et al., 2017; Charoentong et al., 2017; Danaher et al., 2017; Finotello and Trajanoski, 2018; Thakur et al., 2022). Among these tools, GSVA has been widely used in tumor (Charoentong et al., 2017; Deng et al., 2019; Shen et al., 2019; Xiao et al., 2020; Gong et al., 2021; Zhuang et al., 2021) and non-tumor researches (Hu et al., 2021; Shen et al., 2021; Yu et al., 2021) for core module identification at the gene-set level. AI-based models constructed using low-dimensional biological pathway data generated by GSVA as inputs have become popular and demonstrate promising effects (Chawla et al., 2022; Martinez et al., 2022). However, the application of GSVA-derived core immunosignals with even lower dimensionality for efficient feature selection, which benefits machine learning and deep learning in precision oncology, has not been researched.

In this study, immunogenomic profiling of liver transcriptomes was performed to explore the core immunosignals involved in the entire disease course of hepatitis B and their extended clinical applications in early diagnosis, prognostic assessment, and precision usage of anti-cancer drugs. First, we employed GSVA to identify a meaningful HBV pathogenic gene module, named CLST. The potential role of CLST in predicting liver injury and detecting HBV-LF was uncovered. Co-enrichment of CLST and activated CD4+T cells (aCD4) in liver tissue from HCC patients was identified and experimentally verified in our in-house RNA-seq data. Next, a high enrichment score for nutritional utilization of amino acid-related genes was demonstrated as a predictive factor for poor overall survival (OS). The link between nutritional utilization of amino acids and CLST/aCD4 dysregulation in patients with HBV-HCC was explored. Powerful and explainable machine learning methods were then incorporated to construct tools for tumor tissue identification. Simultaneously, a novel deep residual neural network model (GeneSet-ResNet) based on CLST and aCD4 was proposed for long OS(LS) status prediction. Finally, the utility of aCD4 and CLST for evaluating anti-HCC drug sensitivity was evaluated. A new strategy for the construction of novel gene set-based AI models will be helpful for precision medicine.

## Materials and methods

### Raw data collection and proceeding

A total of 11 Gene Expression Omnibus (GEO) datasets were downloaded from the GEO database (https://www.ncbi.nlm.nih.gov/geo/). The CHCC cohort comprising Chinese patients with HBV-HCC was obtained from NODE (https://www.bio.sino.org/node). The TCGA-LIHC cohort, consisting of HCC patients, was collected from The Cancer Genome Atlas (TCGA). Brief information about the 13 cohorts and workflow of this study are provided in Additional files (Supplementary Table S1; Supplementary Figure S1). R Studio (Version 1.4.1103) was used to obtain raw data (normalization, gene ID convention, clinical information collection) based on the recommended R packages. The CHCC-GSE14520 dataset comprising 396 tumor tissue samples from HBV-HCC patients was cross-technology combined. The non-biological effects across CHCC and GSE14520 were corrected through “SVA” R package (Tang et al., 2021).

### Collection and sequencing of liver cancer tissue

Fresh liver cancer tissue specimens from HBV-HCC patients surgically resected from the Shanghai Public Health Clinical Center affiliated with Fudan University (SPHCC) were collected, aliquoted, and stored in a liquid nitrogen tank at −80°C within 2 h. Total tissue RNA was extracted and sent for transcriptome high-throughput sequencing (RiboBio Co., Ltd.) to compare changes in the transcript mRNA levels of related genes in liver cancer.

### Identification of differentially expressed genes (DEGs)

Grading (G) and staging (S) systems have been utilized for the efficient evaluation of inflammation and fibrosis in chronic liver diseases, respectively. DEGs (S1/S0, S2/S0, S3/S0, and S4/S0) of GSE84044 were downloaded from the supplementary materials provided in a previous study (Wang et al., 2017) and visualized using GraphPad Prism. DEGs (G1/G0, G2/G0, G3/G0, and G4/G0) of GSE84044 were screened primarily *via* “Limma” R package and visualized *via* “ggplot2” R package, “pheatmap” R package or “EnhancedVolcano” R package. As for the “Enhanced Volcano” R package, upregulated genes with fold change (FC) > 1.5 and *p*-value < 0.05 were considered statistically significant. Venn analysis was used to identify overlapping DEGs.

### Functional annotation and hub genes screening

Gene Ontology (GO) analyses were performed to investigate the biological function annotation of overlapping DEGs of GSE84044 using “clusterProfiler” R package and visualized *via* the “ggplot2” R package. Kyoto Encyclopedia of Genes and Genomes (KEGG) signaling pathway analyses were based on “clusterProfiler” R package and also visualized *via* the “ggplot2” R package. A PPI network of overlapping DEGs from GSE84044, containing 57 nodes and 89 edges, was constructed using the STRING database. Cytoscape software was used to visualize and screen the hub genes. Protein and protein interaction (PPI) analyses of member genes of aCD4 were conducted and visualized using online tools provided by the STRING database.

### ssGSEA score calculation

The enrichment scores (ES) of 28 LILs and CLST in liver samples from the GEO database or NODE were calculated primarily *via* the “GSVA” R package with single sample gene set enrichment analysis (ssGSEA) algorithm (Hanzelmann et al., 2013; Charoentong et al., 2017; Yu et al., 2021). A total of 28 gene sets consisting of cell-specific marker genes represent 28 LILs (Charoentong et al., 2017). CLST and amino acid utilization-associated gene signatures were defined in this study according to previous studies (Subramanian et al., 2005; Barbie et al., 2009).

### Correlation and comparison

The heatmap showing spearman comparison among hub genes and grading (or staging) was calculated and drawn by using the “Hmisc” R package. The “Hmisc” R package was utilized to calculate the correlations between selected genes and LILs. The R package “ggcorrplot” was used to calculate correlations between CLST and LILs. The results were visualized using the “pheatmap” R package. Comparisons of differences between the two groups were performed and visualized as box plots or dot plots *via* the “ggplot2” R package, and heatmap *via* the “pheatmap” R package, respectively according to the guidelines. Statistical significance was set at *p* < 0.05.

### Diagnostic values evaluation and overall survival analysis

The diagnostic values of CLST and LILs immune signals for identifying whether CHB patients are living with liver injury or liver fibrosis were calculated through COX analysis using the “pROC” R package based on liver transcriptomes of GSE83148 and GSE84044, respectively. OS analysis was performed using the Kaplan-Meier survival” R package based on expression values of hub genes or ES of identified immunogenomic signals in tumor tissues of GSE14520 and/or CHCC with available survival information. Kaplan-Meier curves were drawn and plotted *via* the “survminer” R package. Statistical significance was set at *p* < 0.05.

### Explainable machine learning algorithms for tumor tissue detection

Nine powerful AI algorithms, including logistic regression (LR), linear discriminant analysis (LDA), K neighbors (KNN), Gaussian naive Bayes (GNB), support vector machine (SVM), random forest (RF), decision tree (CRAT), gradient boosting decision tree (GBDT), and LightGBM (LGBM, leaf-wise GBDT) were evaluated for tumor detection. The area under the curve (AUC) was calculated to quantify predictive performance. Shapley additive explanation method (SHAP) was implemented to provide the model-level quantitative interpretation by evaluating the importance of each feature to the classification.

### Long term OS analysis *via* GeneSet-ResNet

A two-dimensional (2-D) ResNet-18 model, called GeneSet-ResNet, was proposed in this study, where the input layers receiving 2-D pseudo-images were converted by the expression values of unique feature genes of both CLST and aCD4 that could be detected in the liver transcriptomes of HCC patients. The sample imbalance between HCC patients with long-term overall survival (LS) and those with short-term overall survival (SS) was solved using Borderline SMOTE. Repeated stratified K-fold cross-validations (splits = 10, repeats = 30, and random state = 2022) were used in the GeneSet-ResNet model. In each 10-fold cross-validation, the dataset was randomly divided into a training set (70% of the samples) for batch training and a test set (10% of the samples) for performance evaluation. The model performance was also validated using a validation set comprising 20% of the samples. In addition, excellent training results and generalization ability were achieved by employing the root-mean-square propagation (RMsprop) optimization algorithm and the learning rate decay method. Accuracy (ACC) were calculated as follows:

$$ACC=\left(TP+TN\right)/\left(TP+TN+FN+FP\right)$$

TP, true positive; FP, false positive; TN, true negative; FN, false negative.

The area under the curve (AUC) was calculated to quantify predictive performance.

### Chemotherapy sensitivity prediction

The half-maximal inhibitory concentration (IC50) for patients with HCC based on liver transcriptomes was predicted using the “calcPhenotype” algorithm provided by a ridge regression model (“oncoPredict” R package) (Maeser et al., 2021). The differences in sensitivity between first-line and emerging drugs used for HCC treatment between HBV-HCC patients in the CLST ^high^ aCD4 ^high^ subgroup and those in the CLST^low^aCD4 ^low^ subgroup were analyzed using the Wilcoxon test. Statistical significance was set at *p* < 0.05.

## Results

### CLST definition

**FIGURE 1:**
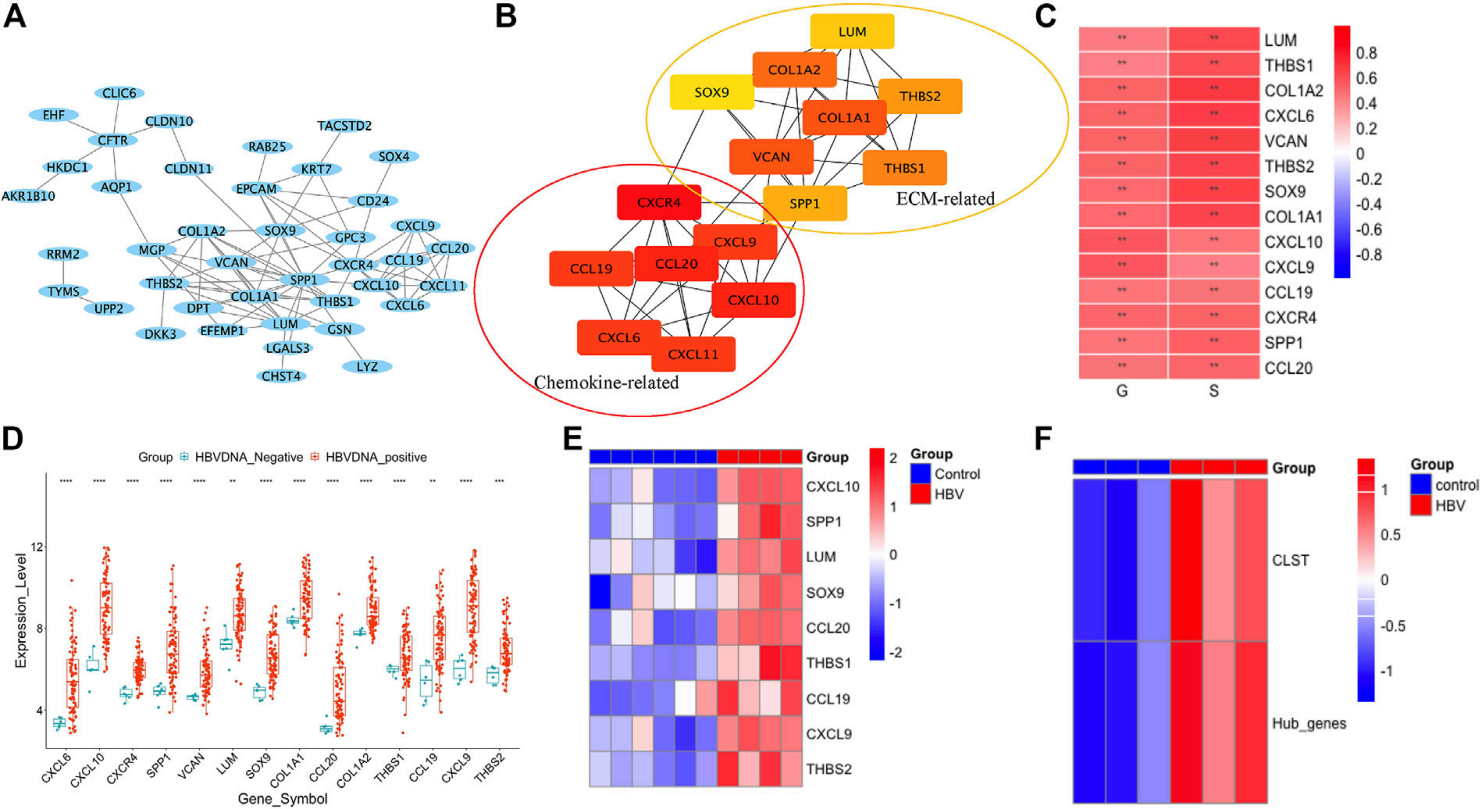
CLST identification. **(A)** PPI network of overlapping DEGs by STRING (GSE84044). **(B)** Seven chemokine-related genes and eight ECM-related genes in two groups were further identified using Cytoscape of MCODE plug-in. **(C)** Correlations among expression values of 14 hub genes, G scores, and S scores. **(D)** Dot plots of 14 GS-associated hub genes in liver samples of CHB group and control group. **(E)** Heatmap of nine hub genes in HBV-infected mice and control mice. **(F)** Heatmap showing ES of gene set comprised of 14 GS-associated hub genes and CLST in HBV-PHH and control PHH.

The gene expression profiles of HBV-LF were re-analyzed according to a previous study. Overlapping DEGs upregulated in S2, S3, and S4 when compared to the S0 group were selected (Supplementary Figures S2A, S2B). Chemokine signaling pathways in which cargo-carrying genes encoding CXC subfamily ligands and CCL subfamily ligands were observed to be primarily enriched (Supplementary Figures S2C, S2D). Of the overlapping DEGs, 15 hub genes belonging to the chemokine-related gene cluster and ECM-related gene cluster with the highest maximal clique centrality (MCC) score were screened (Figures 1A,B). The majority of 15 hub genes were also significantly upregulated in the G2, G3, and G4 groups compared to the G0 group (Supplementary Figures S3A–D). Fourteen hub genes that were positively associated with G and S were listed in this study as GS-associated hub genes (Figure 1C). These genes were confirmed to be upregulated in the liver tissues of HBV-infected patients (Figure 1D) and CHB patients with liver injury (Supplementary Figures S3D−E) compared to normal controls. All GS-associated hub genes were highly enriched in CHB patients at immune active (IA) phases (Liu et al., 2018) and displayed a similar expression pattern in CHB patients at immune tolerance phases (IT) and immune carrier phases (IC) (Supplementary Figure S3F). To further uncover the original inducers of GS-associated hub genes, the liver transcriptomes of HBV-infected human hepatocyte chimeric mice were analyzed. We found that GS-associated hub genes that could be detected in liver tissues of human hepatocyte chimeric mice were upregulated upon HBV infection (Figure 1E) and significantly expanded in *ex vivo* HBV-infected human primary hepatocytes (PHH) (Figure 1F). Therefore, in our study, we defined GS-associated hub genes as a gene set, including CCL19, CCL20, CXCL9, CXCL10, LUM, SOX9, SPP1, THBS1, and THBS2, named CLST.

### CLST, co-expanded with LILs, could effectively predict HBV-liver inflammation and fibrosis

**FIGURE 2:**
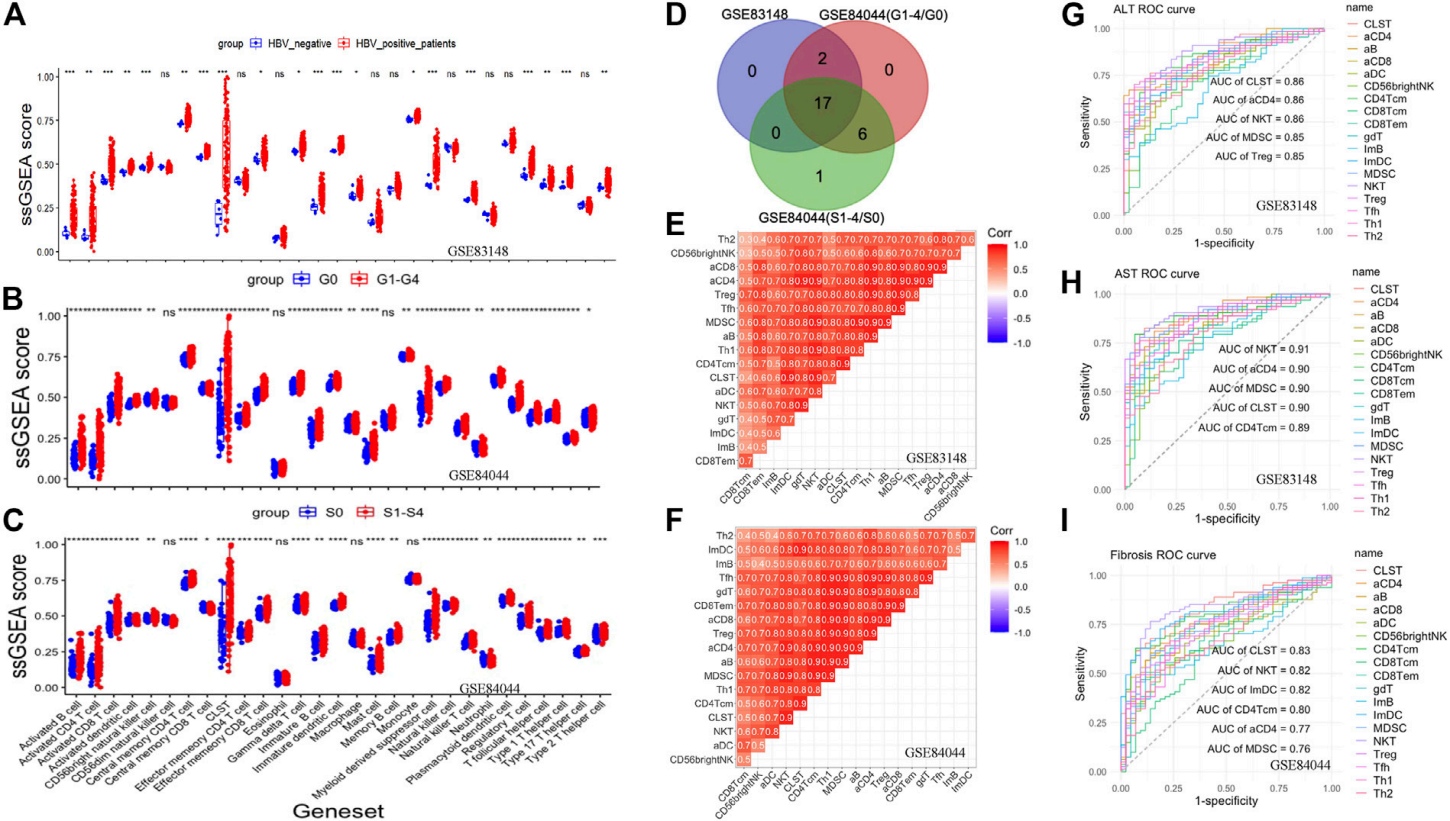
Co-enrichment and diagnostic values of CLST, NKT, MDSC, and aCD4 in CHB and HBV-LF. **(A)** Boxplot comparing immune signals between patients with chronic HBV infection and normal tissues from patients without HBV infection. **(B)** Boxplot comparing immune signals between patients with inflammation (G ≥ 1) and those without inflammation (G = 0). **(C)** Boxplot comparing immune signals between patients with liver fibrosis (S ≥ 1) and those without liver fibrosis (S = 0). **(D)** Venn diagram of upregulated LILs. **(E,F)** Correlation heatmap showing the co-enrichment pattern of CLST and LILs in CHB and HBV-LF. **(G**–**I)** ROC curves of CLST and LILs for predicting liver injury and LF.

The host immune response plays an important role in HBV pathogenesis. Therefore, the landscape of CLST and LILs in CHB and LF is presented in this section. Both CLST and LILs were highly enriched in HBV-infected patients (Figure 2A), CHB patients with a higher score of liver inflammation that was characterized by G (Figure 2B), and with a higher score of LF that was characterized by S (Figure 2C). As shown in Figure 2D, 17 overlapping LILs, including NKT, MDSC, and activated T cells bearing CCL20 (aCD4), were screened from 28 LILs. Overlapping LILs were co-expressed with CLST in liver samples of CHB (Figure 2E) and HBV-LF (Figure 2F). Generally, we can conclude that CLST can be directly induced upon initial HBV infection and is associated with liver inflammation (G) and LF (S). All AUCs of CLST, NKT, MDSC, and activated T cells bearing CCL20 (aCD4) in predicting abnormal serum ALT/AST levels were above 0.85 (Figures 2G,H). Moreover, CLST was ranked as the leading gene set, followed by NKT, aCD4, and MDSC, which effectively segregated LF from normal liver samples (Figure 2I).

### CLST synergizing with aCD4 were risk signals in HBV-HCC

**FIGURE 3:**
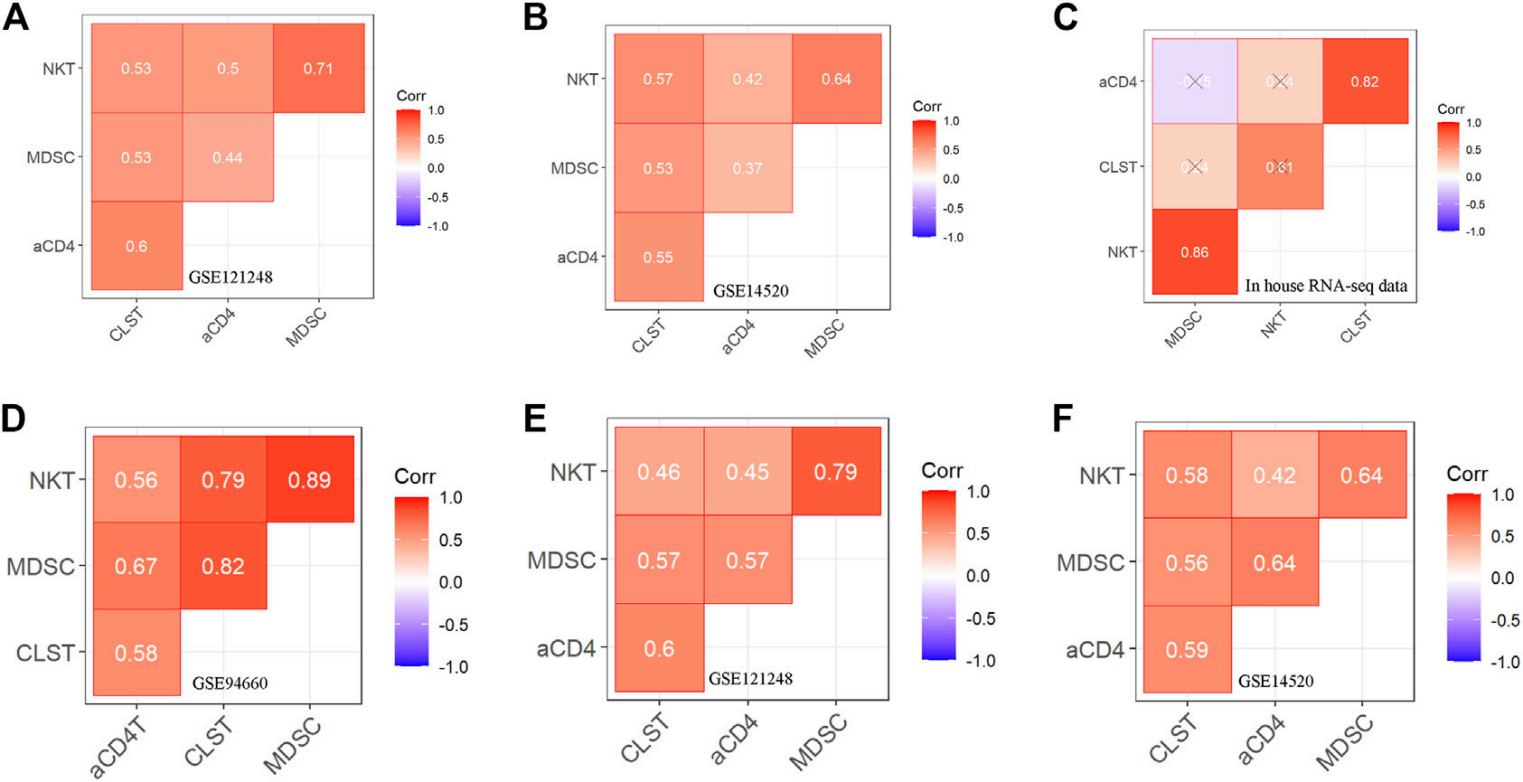
Correlation between CLST and LILs in tumor tissues of patients with HBV-HCC **(A–C)** Pearson correlation analysis showing co-enrichment among CLST, aCD4, NKT, and MDSC in liver tissues of GSE121248, GSE14520, and our in-house RNA-seq data. **(D–F)** Pearson correlation analysis showing co-enrichment among CLST, aCD4, NKT, and MDSC signals in liver tumor tissues of three independent GSE datasets.

**FIGURE 4:**
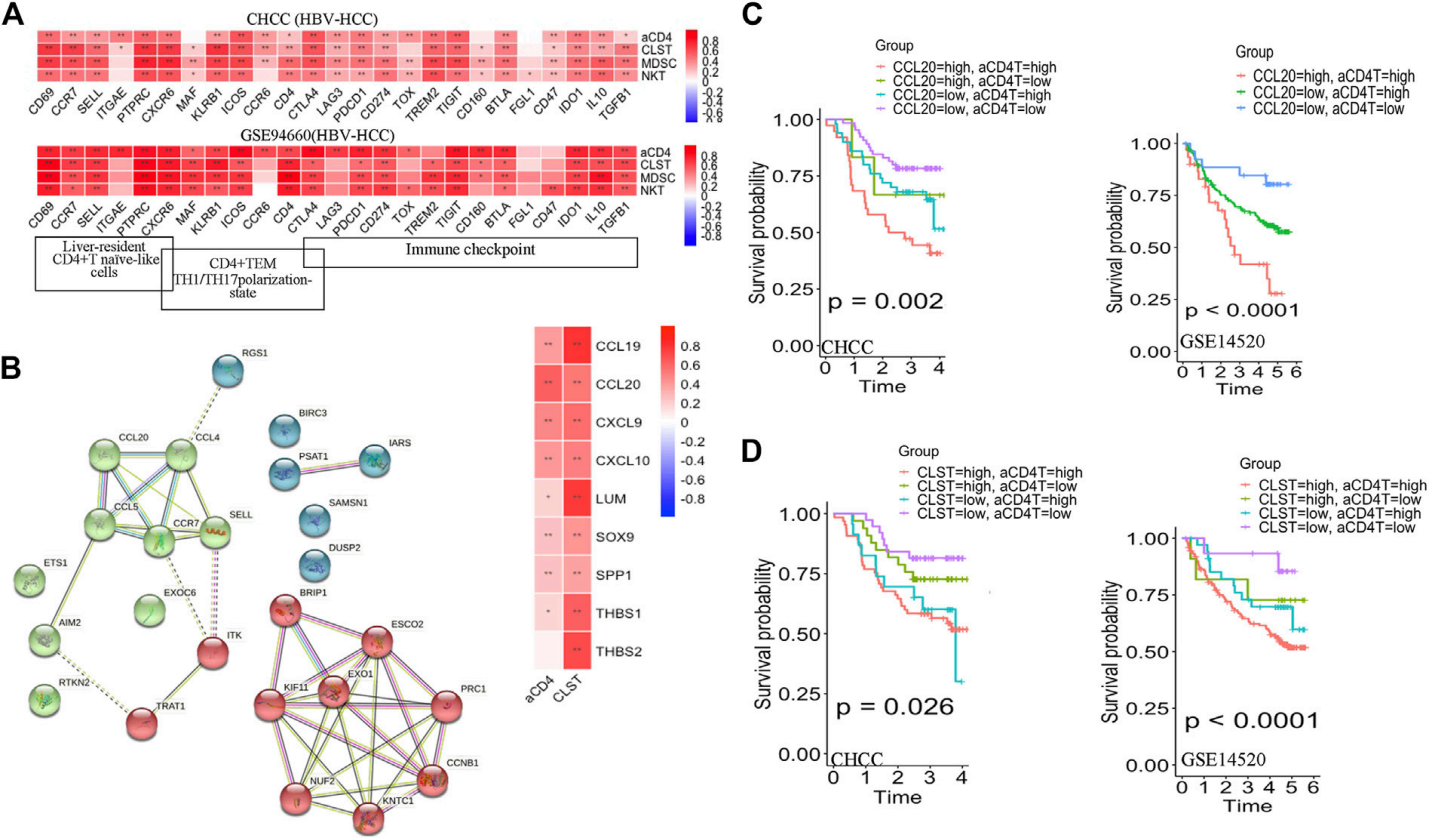
Prognostic values of CLST and aCD4 for OS prediction in HBV-HCC. **(A)** Heatmaps showing correlations between CLST, aCD4, MDSC, NKT, and specific immune genes in HBV-HCC. **(B)** PPI analysis of member genes belonging to aCD4 and correlations among aCD4, CLST, and hub gene expression values. CCL20 was an overlapping gene in both aCD4 and CLST (GSE14520). **(C,D)** KM survival analysis of OS in tumor tissues with a higher ES of both aCD4 and CCL20 or a higher ES of both aCD4 and CLST in two independent HBV-HCC cohorts. Time was calculated in years. The log-rank test for *p*-value and *p*-value <0.05 was considered significant.

The batch effect among GSE83148, GSE84044, and GSE14520 was removed by using the SVA algorithm (Supplementary Figures S4A,B). Enrichment Scores of CLST, NKT, aCD4, and MDSC were identified to be significantly higher in tumor tissues of HBV-HCC than in those without HBV in the integrated gene microarray dataset (Supplementary Figure S4C). Correlation analyses were performed in normal and tumor tissue mixed samples of two independent HBV-HCC cohorts (Figures 3A,B) and our HBV-HCC data (Figure 3C), and severe positive relationships between CLST and aCD4 were verified. In addition, CLST and aCD4 were significantly co-enriched in the tumor tissues of the three independent HBV-HCC cohorts (Figures 3D–F). Interestingly, positive correlations among CLST, liver-resident CD4^+^ T naïve-like cells (CD4+T_LR-NL_), acquisition of a TH17 polarization state (CD4+T_LR-NL_), CD4+T_EM-TH1/TH17_, and immune checkpoints (ICs) indicated their cross-talk in the tumor tissue of HBV-HCC (Figure 4A).

Additionally, PPI analysis revealed that CCL20 was the leading gene exhibiting the closest relationship with aCD4 in HBV-HCC patients (Figure 4B). Further survival analysis suggested that a higher aCD4/CLST/CCL20 was associated with significantly shorter OS (Supplementary Figure S5). The CLST^high^aCD4^high^ (Figure 4C) and aCD4^high^CCL20^high^ (Figure 4D) subgroups showed worse OS probabilities, highlighting the application of CLST and aCD4 for the establishment of diagnostic and prognostic models in HCC patients.

### Patients with HBV-HCC in the CLST^high^aCD4^high^ subgroup were characterized by an unfavorable status of excess nutritional usage of amino acids

**FIGURE 5:**
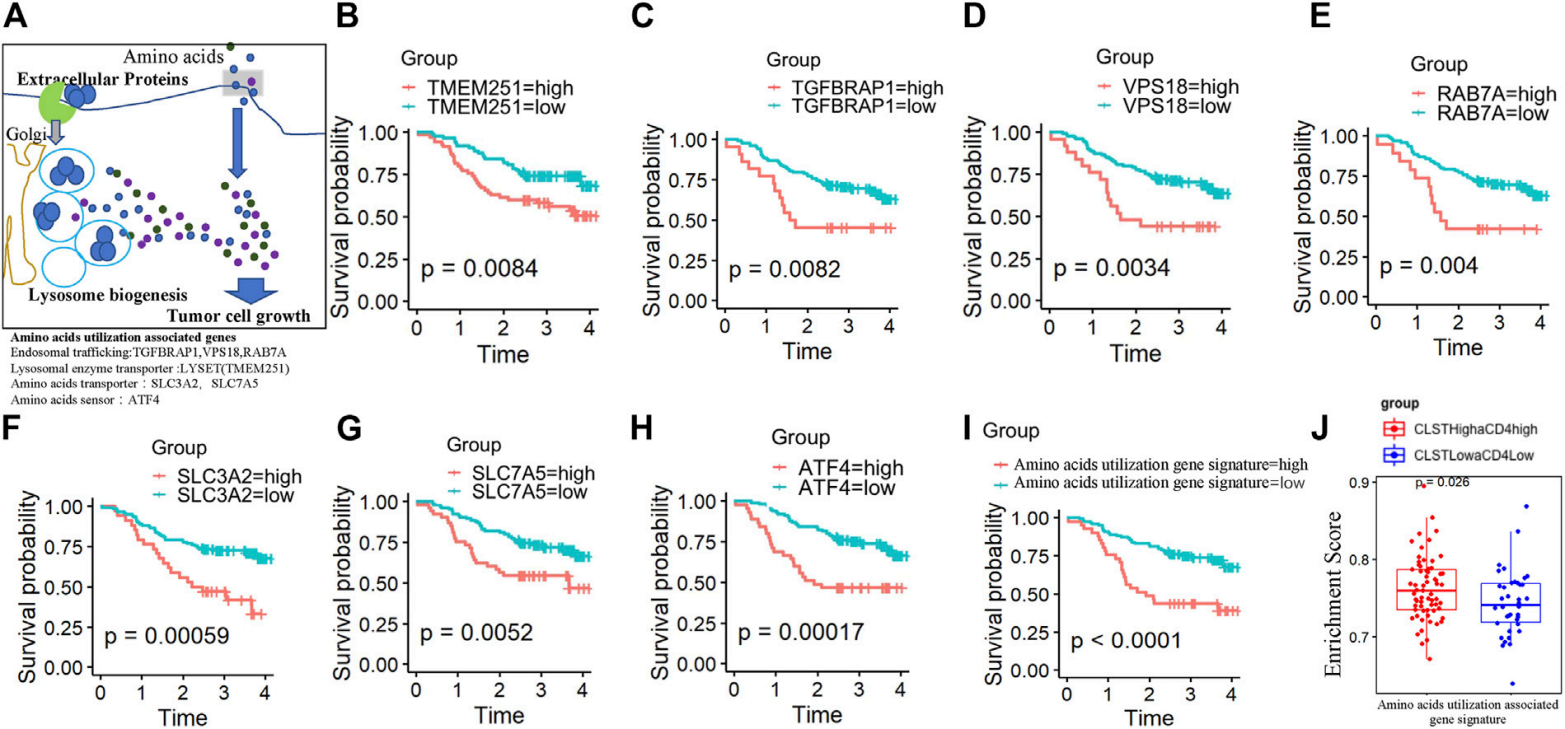
The prognostic value of nutritional utilization of the amino acid-associated gene signature in HBV-HCC **(A)** Lysosomal nutrient generation and nutritional utilization of amino acids for tumor cell growth. LYSET (TEME251), ATF4, VPS18, RAB7A, SLC7A5, TGFBRAP1, and GNPTAB were involved in this process. **(B–H)** Plots depict the KM survival curves for each nutritional utilization of amino acid-associated genes in HBV-HCC patients from the CHCC cohort divided into low and high expression groups according to the gene expression value. **(I)** KM survival curves for OS in tumor tissues of HBV-HCC patients from CHCC cohort with a high ES of the “nutritional utilization of amino acid-associated gene” signature and a low ES of the “nutritional utilization of amino acid-associated gene” signature. **(J)** Differences in the enrichment levels of the “nutritional utilization of amino acid-associated gene” signature between HBV-HCC patients from the CHCC cohort in the CLST^high^aCD4^high^ subgroup and those in the CLST^low^aCD4^low^ subgroup.

As shown in Figure 5A, LYSET, ATF4, VPS18, RAB7A, SLC7A5, TGFBRAP1, and GNPTAB were previously identified as proteins involved in the nutritional utilization of amino acids (Pechincha et al., 2022; Richards et al., 2022). Surprisingly, survival analysis showed that a higher gene expression level of LYSET/ATF4/VPS18/RAB7A/SLC7A5/TGFBRAP1/GNPTAB was associated with a significantly shorter OS in HBV-HCC patients (Figures 5B–H). HBV-HCC patients in the CLST^high^aCD4^high^ and CLST^low^aCD4^low^ subgroups exhibited a distinct pattern of GSVA-based amino acid utilization-associated gene signature. Consistently, a higher ES of the amino acid utilization-associated gene signature represented a worse OS probability (Figure 5I). The ES of amino acid utilization-associated gene signature was found to increase in the CLST^high^aCD4^high^ subgroup, reflecting a shorter OS (Figure 5J).

### An explainable machine learning model based on feature genes belonging to CLST and aCD4 was powerful for tumor tissue detection

**FIGURE 6:**
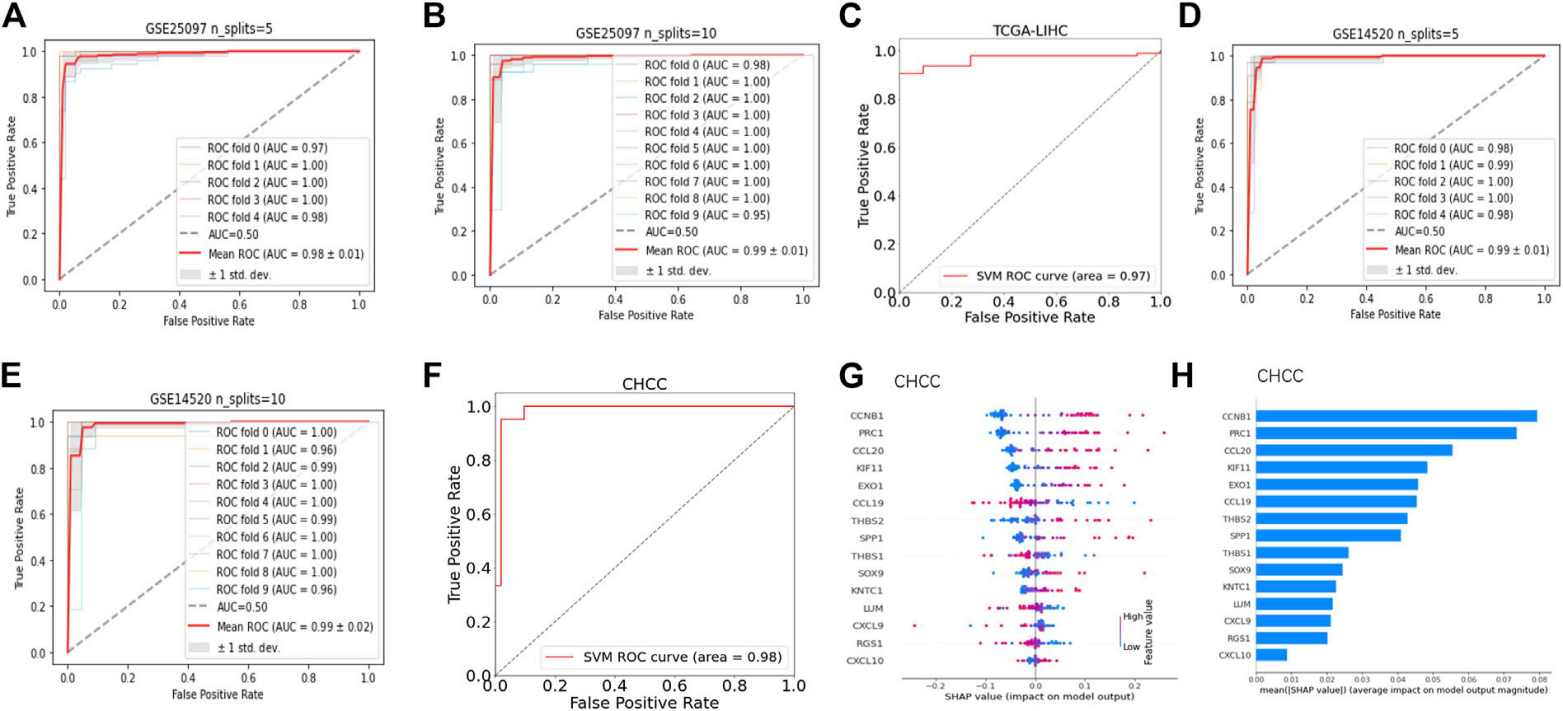
Fifteen feature genes of CLST and aCD4 were promising diagnostic signals for tumor tissue identification. **(A,B)** ROC curves of expression values of 15 feature genes for HCC tumor prediction among HCC tumor, adjacent non-tumor, cirrhotic, and normal liver samples using SVM with stratified K-fold cross-validations (n_splits = 5 and 10). **(C)** ROC curves of expression values of 15 feature genes as a diagnostic set for the separation of HCC tumors from non-tumor liver samples *via* SVM. **(D,E)** AUC of ROC curves of expression values of 15 feature genes as diagnostic markers for early-stage HBV-HCC tumor identification from non-tumor liver samples *via* SVM with stratified K-fold cross-validations (n_splits = 5 and 10). **(F)** ROC curves of expression values of 15 feature genes as diagnostic markers for separation of tumor tissues at the early stage of HBV-HCC from non-tumor liver samples in the CHCC cohort *via* SVM. **(G,H)** SHAP profiles of 15 feature genes of the outperformed SVM model in the CHCC cohort. The dot plot shows the effect of the expression value of the feature gene on the model output. The bar plot shows the decreasing average feature importance of the expression value of the 15 feature genes on the influence of the final model output for predicting tumor tissues at an early stage.

Nearly half of the feature genes belonging to aCD4 at higher levels were associated with a significantly shorter OS in the CHCC cohort (Supplementary Figure S6). Among them, seven genes (KIF11, CCNB1, EXO1, KNTC1, PRC1, RGS1, and CCL20) were identified as overlapping risk factors for survival in the GSE14520 cohort (data not shown). Thus, fifteen feature genes comprised of nine genes from CLST and seven genes from aCD4 were ultimately used to construct a diagnostic model for tumor tissue detection. Briefly, nine AI algorithms were trained and validated to separate tumor tissues from the normal liver, cirrhosis, and tumor tissues in the GSE25097 cohort. Of the nine AI algorithms, SVM outperformed in terms of the highest ACC (Supplementary Figure S7A), showed potent robustness with stratified K-fold cross-validations, and achieved the highest average AUC that could accurately separate tumor tissue from any other type of liver sample (Figures 6A,B). The efficiency of SVM was further tested in an independent HCC cohort (TCGA-LIHC), with an AUC of 0.97 (Figure 6C). SVM also was powerful in separating tumor tissues at early stage (BCLC stage 0-A) from non-tumor tissues (GSE14520) among nine AI algorithms (Supplementary Figure S7B) and achieved an average AUC of 0.99 and 0.99 with stratified K fold cross-validations (splits = 5 and 10), respectively (Figures 6D,E). The diagnostic power of SVM was also excellent in an independent test set (CHCC), with an AUC of 0.98 (Figure 6F). The SHAP summary plot suggested that CCNB1, PRC1, CCL20, KIF11, and EXO1 were the top five variables that had important impacts on the performance of SVM in the CHCC cohort (Figures 6G,H).

### Deep learning model fed by feature genes from CLST and aCD4 was efficient for LS prediction

**FIGURE 7:**
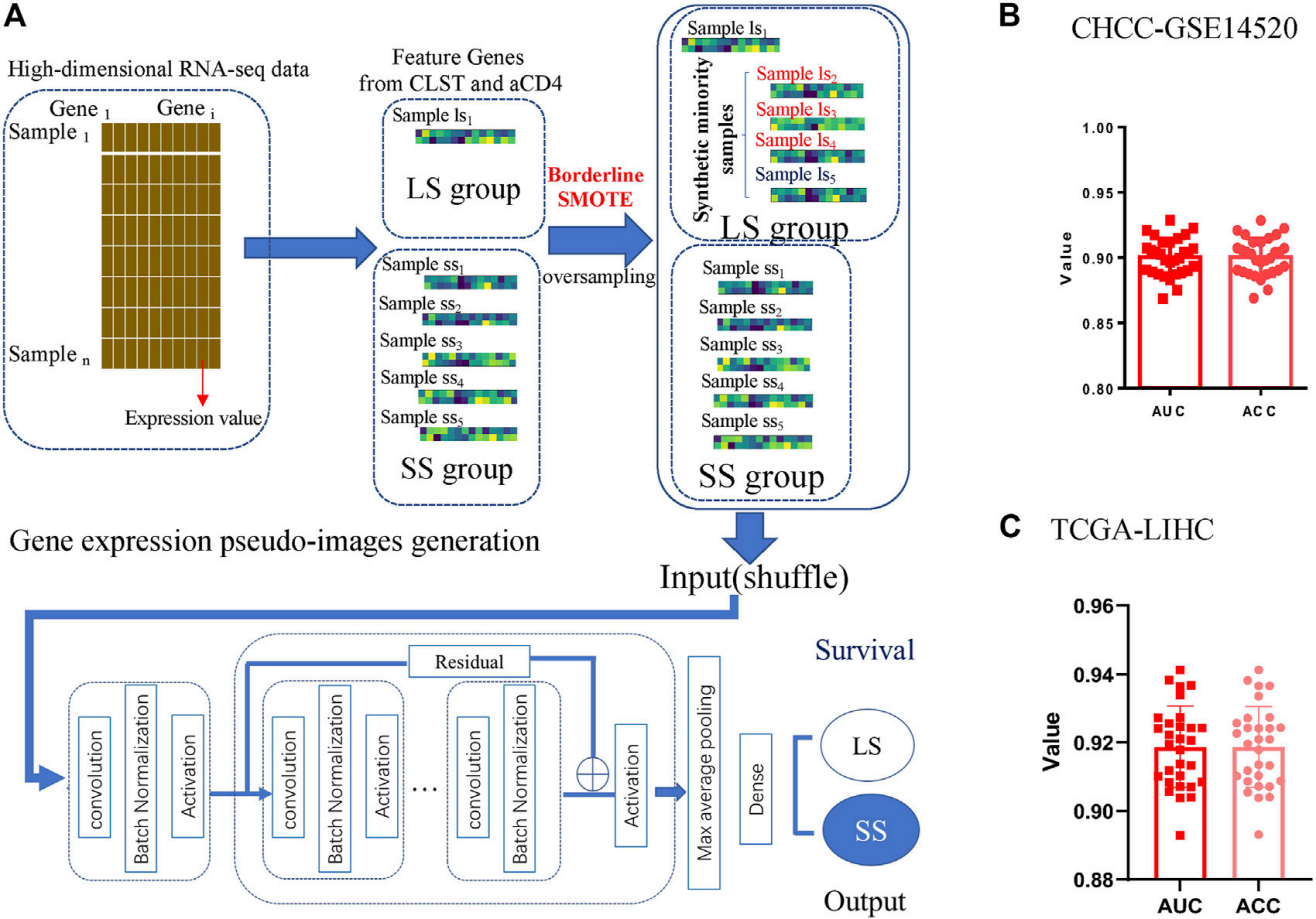
Unique feature genes belonging to CLST and aCD4 were promising prognostic signals in LS prediction. **(A)** Process of LS prediction in HCC patients (generation of gene expression pseudo-images with CLST and aCD4, oversampling with synthetic minority samples, input layer, detailed architecture of the deep residual network module, and output layer for LS status prediction). **(B)** The average AUC and ACC values of 30 repeats for LS status (>5 years) prediction in HBV-HCC patients. **(C)** The average AUC and ACC values of 30 repeats for LS status (>5 years) prediction in HCC patients.

The process of generating gene expression pseudo-images and the GeneSet-ResNet architecture using resnet-18 as the backbone for LS prediction is illustrated in Figure 7A. In brief, there were 26 small squares (rows = 2, columns = 13) in each pseudo-image representing the expression value of 26 unique feature genes from one HBV-HCC sample. The sample imbalance between the LS and SS subgroups was solved using borderline SMOTE generated synthetic minority samples. The LS and SS subgroups in HBV-HCC were further classified using the GeneSet-ResNet model with gene expression pseudo-images as inputs. Model performance was evaluated in 30 repeated stratified 10-fold cross-validations. As shown in Figure 7B, an average AUC of 0.907 and ACC of 0.919 over 30 repeats of the stratified 10-fold cross-validation for LS (survival time >5 years) prediction were achieved in the CHCC-GSE14520 dataset. Interestingly, the GeneSet-ResNet model outperformed the TCGA-LIHC dataset in LS prediction (Figure 7C). These results suggest that GeneSet-ResNet, based on CLST and aCD4, is a robust deep learning model for 5 years LS prediction in HCC.

### CLST and aCD4 guided precision anti-HBV immunotherapy and anti-cancer chemotherapy

**FIGURE 8:**
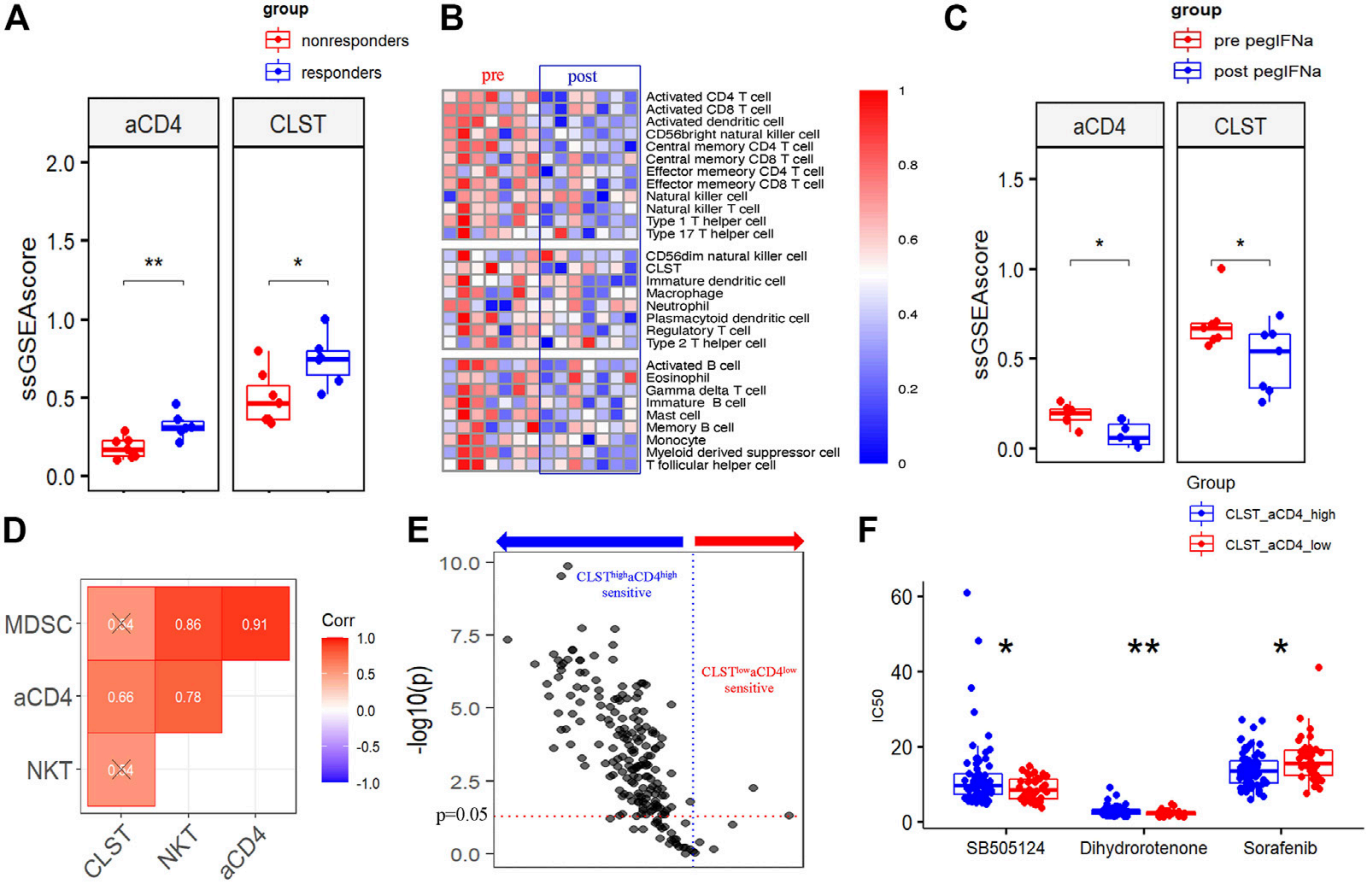
CLST and aCD4 were involved in drug sensitivity to anti-HBV immunotherapy and anti-cancer chemotherapies. **(A)** Comparisons of CLST and aCD4 between PEG IFN-α treatment responders and non-responders (GSE27555). **(B)** Heatmaps showing differences in liver samples from HBV-infected patients pre and post PEG-IFN-α treatment (GSE66698). **(C)** Boxplot of pairwise comparisons of CLST, aCD4, NKT, and MDSC between the control group and PEG IFN-α-treated group (GSE66698). **(D)** Correlations among CLST, aCD4, NKT, and MDSC in PEG IFN-α-treated liver samples (GSE66698). **(E)** Volcano plot of the sensitivity of HBV-HCC patients in the CLST^high^aCD4^high^ subgroup and CLST^low^aCD4^low^ subgroup to 198 anti-cancer drugs. **(F)** Comparisons of the sensitivity to first-line chemotherapy (sorafenib) and emerging chemotherapies (SB505124, dihydrorotenone) between the CLST^high^aCD4^high^ subgroup and CLST^low^aCD4^low^ subgroup (CHCC).

The ESs of CLST and aCD4 in liver transcriptomes from a CHB cohort treated with IFN-α therapy were calculated, and the results indicated that CLST and aCD4 were remarkably upregulated in treatment responders (Figure 8A). These results suggest that the sensitivity of anti-HBV immunotherapy can be predicted using CLST and aCD4. The ESs of CLST and 28 LILs in the liver tissues of treatment responders pre- and post-IFN-α were also evaluated. CLST and LILs tended to be downregulated in responders after receiving PEG IFN-α (Figure 8B). Both CLST and aCD4 were significantly suppressed in paired samples with the engagement of PEG IFN-α (Figure 8C). Moreover, only aCD4 and CLST levels showed a significant positive correlation in these responders (Figure 8D). The sensitivities of the CLST^high^aCD4^high^ and CLST^low^aCD4^low^ subgroups in HBV-HCC patients to 198 anticancer chemotherapies from a resource for therapeutic biomarker discovery in cancer cells (Genomics of Drug Sensitivity in Cancer, GDSC) were compared (Supplementary Table S2; Figure 8E). HBV-HCC patients in the CLST^high^aCD4^high^ subgroup were more sensitive to the majority of anticancer drugs (167/198) than those in the CLST^low^aCD4^low^ subgroup (Figure 8E). In terms of first-line chemotherapy selection, patients in the CLST^high^aCD4^high^ subgroup were more sensitive to sorafenib (Figure 8F). Patients in the CLST^low^aCD4^low^ subgroup were more sensitive to two emerging chemotherapies: SB505124 (TGF-β receptor inhibitor) and dihydrorotenone (Figure 8F).

## Discussion

Although most of the feature genes in CLST, including intrahepatic mRNA for CXCL9 (Wang et al., 2017; Jiang et al., 2021), CXCL10 (Wang et al., 2017; Singh et al., 2020; Jiang et al., 2021), CCL20 (Zhao et al., 2014), SOX9 (Xu et al., 2016; Yang et al., 2020), SPP1 (Shang et al., 2012), and LUM (Xu et al., 2016) have been reported involved in several HBV-related diseases, there are no reports systemically describing their landscape during all the stages of HBV related diseases even less the integration of these genes as a gene set for predicting liver injury and LF, to our best knowledge. Host-encoding genes can serve as prognostic biomarkers in LF, and a fibrosis risk score (FRS) has been established; however, none of these studies considered global immunogenomic information in consideration (Xu et al., 2016; Zhou et al., 2017; Singh et al., 2018). “GSVA” R package is a powerful tool for analyzing and exploring the complex involvement of the immune microenvironment in larger samples (Moeini et al., 2019; Chawla et al., 2022; Martin-Serrano et al., 2022). Through GSVA, we identified hub genes associated with HBV pathogenicity and demonstrated that the CLST signal initially induced by HBV infection was co-enriched with the majority of LILs in CHB and HBV-LF patients. CLST was ranked as the leading factor for efficient diagnosis of CHB patients living with LF compared to those without LF. Interestingly, CLST and aCD4 exhibited the strongest correlation in the largest HBV-HCC cohort among multiple independent cohorts and were verified in our in-house HBV-HCC patients. These observations suggest that the CLST-aCD4 axis plays an important role in HCC pathogenesis. Mechanistically, CLST and aCD4 were found to be highly associated with both Th1/Th17 polarization and ICs in tumor tissues.

TH17 has been widely reported to be an important inflammatory factor in HCC (Bansal, 2020; Ma et al., 2020; Li et al., 2021). Recently, the expansion of liver-resident CD4+T naïve-like cells (CD4+T_LR-NL_) acquiring a TH17 polarization state has been proven to be a candidate contributor to primary sclerosing cholangitis (PSC) pathogenesis (Poch et al., 2021). Immune checkpoints (ICs) are associated with poor clinical outcomes in HCC (Ma et al., 2019; Wang et al., 2019; Shen et al., 2022). Interestingly, in this study, positive correlations among CLST, CD4+T_LR-NL_, CD4+T_EM-TH1/TH17_, and ICs indicated their crosstalk in the tumor tissue of HBV-HCC. Th17 cells recruited *via* the CCL20-CCR6 axis in the tumor microenvironment (TME) are drivers of worse clinical outcomes (Zhang et al., 2009; Liao et al., 2013; Li et al., 2016; Li et al., 2017) and ICs have been well demonstrated to account for immunosuppressive microenvironment formation that favors anti-tumor immune evasion (Sangro et al., 2021). Our study leads to the hypothesis that CLST and aCD4 bearing CCL20 are important causes of damaged immune surveillance and TME generation. Actually, a recent study provides a solid foundation for the association between CCL20 and TME and it will be promising for further study in HBV related diseases (Fan et al., 2022). Correspondingly, a higher ES of CLST or aCD4 implies a shorter OS. We provide insights into the 25 member genes of aCD4 and highlight that nearly half of these genes are significantly associated with worse survival rates. Obviously, aCD4 could be referred to as a special CD4+T cell subset at the station of activation. Currently, novel functional immune subsets at the single-cell level resolution have been studied (Song et al., 2020; Lian et al., 2022) and the definition of aCD4 in HBV-related diseases is worthy of further exploration.

Further research in this study also highlights that HBV-HCC patients with dual higher ES of both CLST and aCD4 predict worse overall survival. To uncover the underlying mechanism, we focused on the characteristics of the amino acid utilization system in CLST^high^aCD4^high^ and CLST^low^aCD4^low^ subgroups. Non-glucose nutrients, such as amino acids, lactate, acetate, and macromolecules, can also be absorbed by cancer cells as alternative energy sources (Kamphorst et al., 2015; Pechincha et al., 2022). Both the macropinocytosis and lysosomal catabolic signaling pathways in malignant tumor cells are activated in nutrient-deficient environments (Commisso et al., 2013; Kamphorst et al., 2015; Palm et al., 2015; Pechincha et al., 2022). The increased activity of extracellular protein uptake and lysosomal breakdown constitute an alternative source of amino acids that enables cancer cell growth (Pechincha et al., 2022). Interestingly, we found that each amino acid utilization-associated gene represents a risk factor that affects the clinical outcome of HBV-HCC patients. A lower ES of amino acid utilization associated gene signature in HBV-HCC patients is beneficial for improving survival. We propose that unfavorable nutritional utilization of amino acids may be a potent carcinogenic factor for HCC progression, and the potential link between excess amino acid usage and a dysregulated immune microenvironment according to CLST and aCD4 still requires further experimental exploration.

Dual higher ES of both CLST and aCD4 was critical for the poor progression of HBV-HCC, implying the potential role of CLST/aCD4 interaction in promoting poor clinical outcomes. To test the potential value of CLST and aCD4 in the construction of prognostic models, we present a methodology to compare survival rates for the first time. The survival-sensitive deep residual neural network model based on these two gene sets, named GeneSet-ResNet, outperformed the deep residual neural network classifier in 5 years of LS prediction in liver cancer. This model takes the expression values of low-dimensional feature genes belonging to immunogenomic gene sets as inputs. The gene expression pseudo-images generated in this study were simpler than ever (Hao et al., 2018; Oh et al., 2021; Wang et al., 2022) and hold promising predictive values, thus providing a perspective on their future use in other cancer types.

Fifteen feature genes from CLST and aCD4 were incorporated to perform nine AI algorithms with K-fold cross-validation to detect tumor tissues in HCC. The SVM-derived model was built and worked robustly with high accuracy and a powerful AUC in both training cohorts and independent test cohorts. Our bioinformatics analysis indicated that CLST and aCD4 are powerful diagnostic and prognostic signals across all stages of HBV infection that are suitable for constructing AI models in HCC. There is an urgent need for robust tools to detect tumors at an early stage and predict tumor-related death due to the limitation of efficient HCC treatments, and the AI models developed in this study will facilitate the improvement of clinical management and precision medicine.

PEG IFN-α treatment has the potential to prevent advanced HBV-LF and HBV-HCC occurrence in responders (Liang et al., 2016; Ye and Chen, 2021). The liver transcriptomes of HBV patients receiving standard PEG IFN-α were analyzed to test whether first-line therapy exerts an anti-HBV effect by modulating CLST and aCD4 signals. Correspondingly, CLST and aCD4 were significantly suppressed in responders to PEG IFN-α. These findings suggest that the impact of PEG-IFN-α on improving liver function and inhibiting disease progression during HBV infection is closely related to the CLST-aCD4 axis, which requires further experimental verification. HCC is resistant to current therapies (Song et al., 2021; Rai and Mukherjee, 2022; Xia et al., 2022; Zhang et al., 2022), and a novel strategy that considers the immunology of the disease to improve treatment remains important (Donne and Lujambio, 2022; Rai and Mukherjee, 2022; Shen et al., 2022). Significant differences in sorafenib response between the CLST^high^aCD4^high^ and CLST^low^aCD4^low^ subgroups illustrated that CLST and aCD4 might be important biomarkers for optimizing the use of multi-kinase inhibitors for precision HCC treatment. More importantly, TGF-β inhibition therapies may constitute a promising option for treating HCC in the future. Employing the CLST-aCD4 signal as a predictor allows the appropriate selection of HCC patients that could benefit from interrupting the TGF-β/TβR signaling pathway.

In conclusion, *via* GSVA and AI, our study provides a comprehensive understanding of immune microenvironment-related gene characteristics involved in HBV infection and detect subtle clues for clinical management of HBV-related HCC, providing basis for precision medicine.

There are still limitations in our current study. Although a large number of web accessible high throughput data were enrolled in this study, more experiments are needed for further validation before clinical application of CLST/aCD4 signals in HBV related diseases. The current study only focuses on the clinical application of immunosignals in precision medicine of HBV-related liver diseases, and their specific in HCC at pan-cancer level are promising in further research.

## Data Availability

The original contributions presented in the study are included in the article/Supplementary Material, further inquiries can be directed to the corresponding authors.

## Supplementary material

SUPPLEMENTARY FIGURE S1 Detailed steps of the study.

SUPPLEMENTARY FIGURE S2 Venn diagram and functional annotation of DEGs in HBV-LF. **(A)** Histogram bar chart of upregulated DEGs at differential S were drawn using GraphPad Prism (GSE84044). **(B)** The Venn diagram of overlapping upregulated DEGs among S2/S0, S3/S0, and S4/S0 performed by online tool http://bioinformatics.psb.ugent.be/webtools/Venn/. **(C)** Visualization of GO analysis of 64 DEGs and extracellular matrix (ECM) with the most counts and lowest p value. MF: molecular function. CC, cellular component; BP,biological process. **(D)** Visualization of KEGG pathway analysis. Chemokine signaling pathway was highly enriched.

SUPPLEMENTARY FIGURE S3 Validation of GS associated hub genes in HBV-LF and CHB. **(A-C)** Volcano plots illustrate hub genes that upregulated in G2, G3, and G4 when compared to the G0 group (GSE84044). **(D,E)** Volcano plot performed by “EnhancedVolcano” R package showing 14 GS associated hub genes were upregulated in HBV patients with abnormal ALT and AST when compared to HBV patients with normal ALT and AST, respectively (GSE83148). **(F)** Heatmap of GS associated hub genes in CHB patients at IC, IT, and IA phases (GSE65359).

SUPPLEMENTARY FIGURE S4 Comparisons of immune signals in tumor tissues with HBV and normal tissues without HBV. “SVA” R package was utilized to merge and normalize three microarray datasets (GSE83148, GSE84044, and GSE14520). Batch effects were visualized using PCA algorithm before **(A)** and after removing **(B)** *via* the ComBat function provided by “SVA” R package. **(C)** Boxplot of comparisons of immune signals in HBV-tumor tissue samples and normal samples without HBV infection in combined data.

SUPPLEMENTARY FIGURE S5 Kaplan-Meier (KM) survival analysis. Kaplan-Meier (KM) survival plot for patients according to aCD4 and CLST in HCC (GSE14520).KM analysis of CCL20 in tumor tissues from HBV-HCC patients for OS in two independent cohorts (CHCC, left; GSE14520, right).

SUPPLEMENTARY FIGURE S6 OS analysis of feature genes belonging to aCD4. The association between each of feature genes belonging to aCD4 and OS probability in HBV-HCC tumor tissues (CHCC). Time was calculated by year.

SUPPLEMENTARY FIGURE S7 SVM outperformed in nine AI algorithms for HCC tumor tissue identification. **(A)**Comparison of nine AI algorithms based on expression value of 15 feature genes as a prognostic set with ACC calculation for HCC tumor identification among HCC tumor, adjacent non-tumor, cirrhotic and healthy liver samples (GSE25097). **(B)** Comparison expression value of fifteen feature genes based on nine AI algorithms with ACC calculation for predicting tumor tissue at early stage of HCC (GSE14520).
