# Supplementary material for: Hepatitis B virus pathogenesis relevant immunosignals uncovering amino acids utilization related risk factors guide artificial intelligence-based precision medicine
Source: Front Pharmacol. 2022 Dec 9;13:1079566. doi: 10.3389/fphar.2022.1079566 (PMC9780394; doi:10.3389/fphar.2022.1079566)
Supplement: Supplementary file 1 [file Presentation1.PDF]

Figure S1 Step I

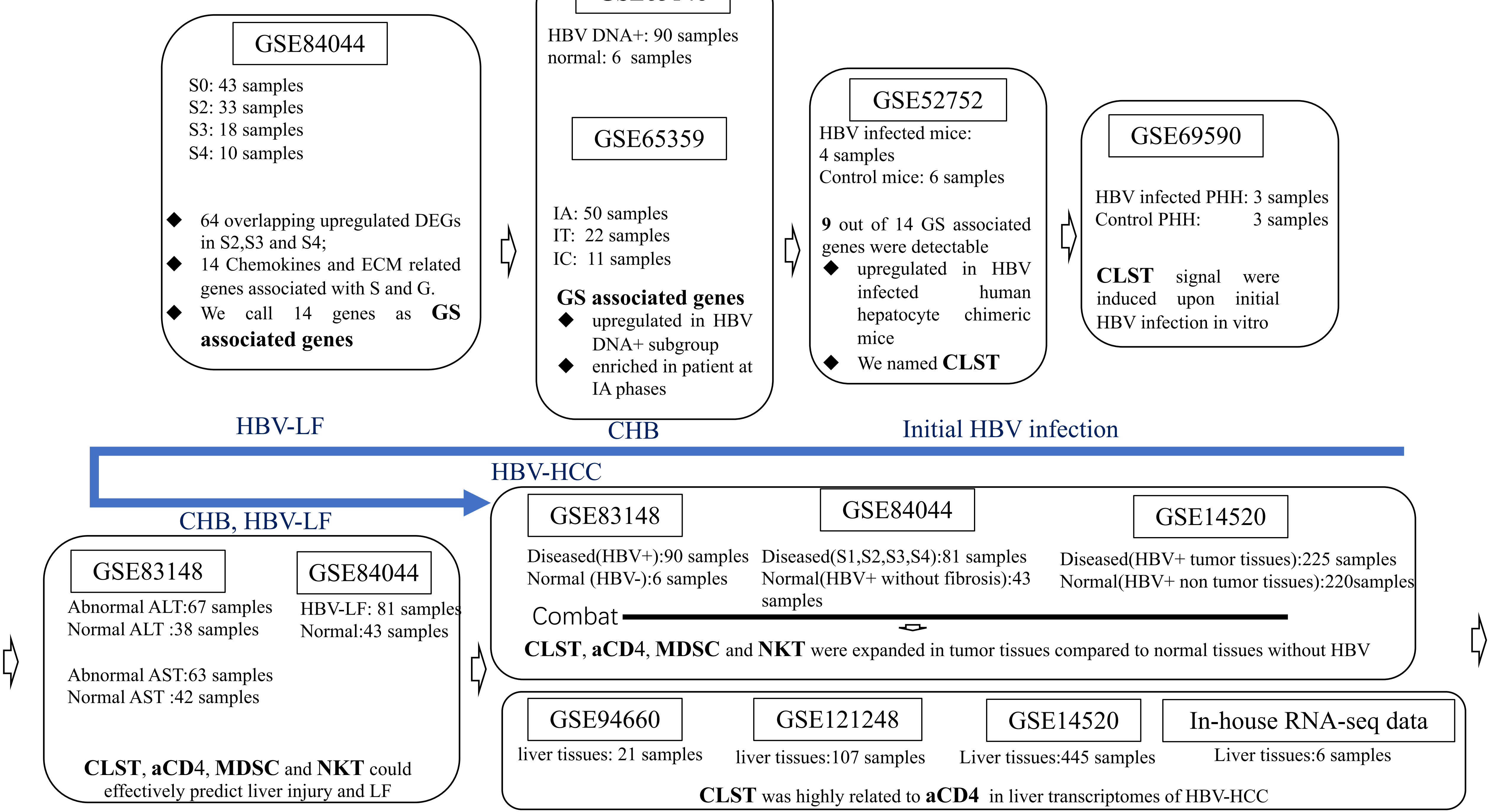

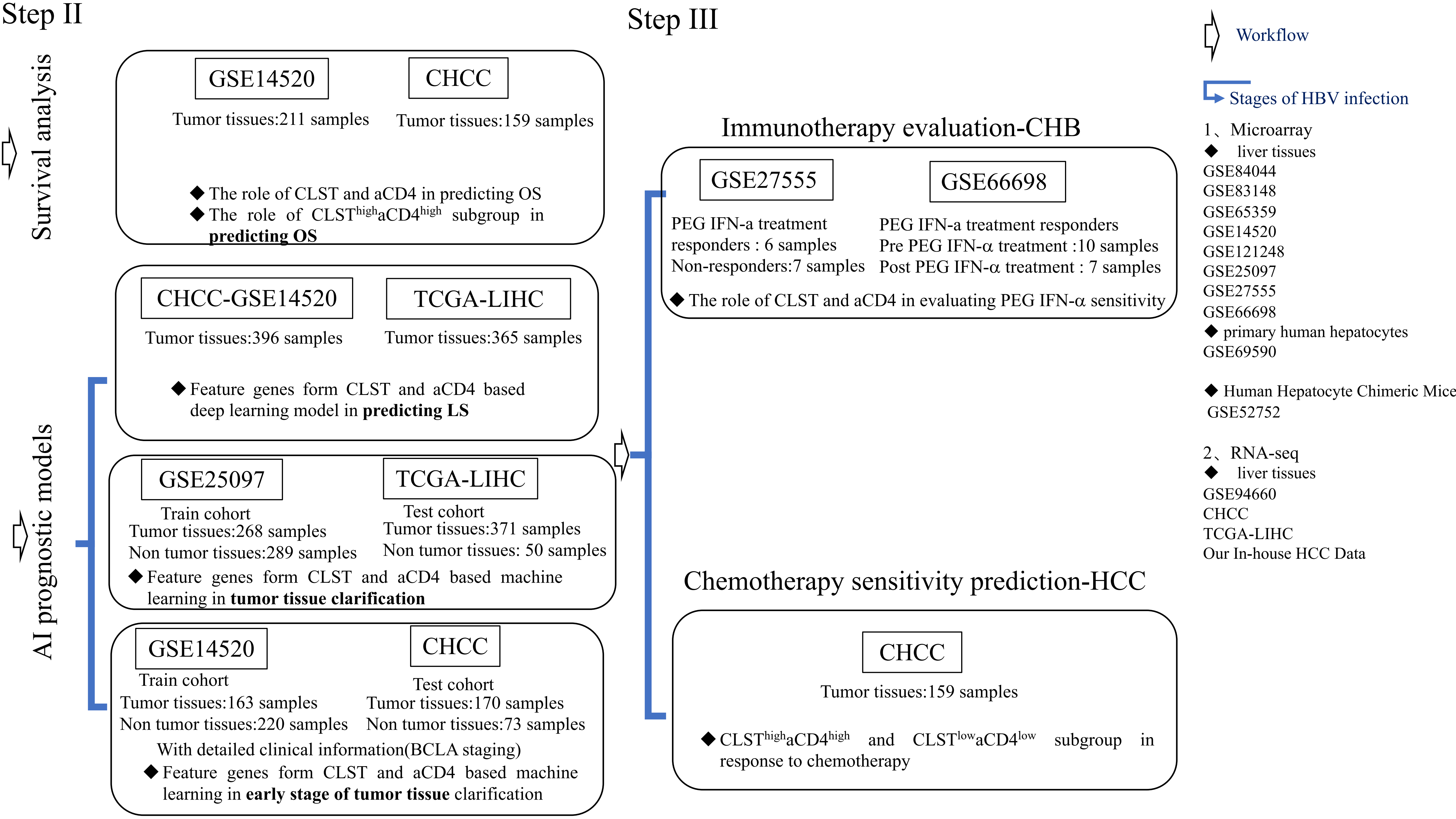
Step II
Survival analysis
GSE14520
Tumor tissues:211 samples
CHCC
Tumor tissues:159 samples
◆ The role of CLST and aCD4 in predicting OS
◆ The role of CLST^high aCD4^high subgroup in **predicting OS**
AI prognostic models
CHCC-GSE14520
Tumor tissues:396 samples
TCGA-LIHC
Tumor tissues:365 samples
◆ Feature genes form CLST and aCD4 based deep learning model in **predicting LS**
GSE25097
Train cohort
Tumor tissues:268 samples
Non tumor tissues:289 samples
TCGA-LIHC
Test cohort
Tumor tissues:371 samples
Non tumor tissues: 50 samples
◆ Feature genes form CLST and aCD4 based machine learning in **tumor tissue clarification**
GSE14520
Train cohort
Tumor tissues:163 samples
Non tumor tissues:220 samples
CHCC
Test cohort
Tumor tissues:170 samples
Non tumor tissues:73 samples
With detailed clinical information(BCLA staging)
◆ Feature genes form CLST and aCD4 based machine learning in **early stage of tumor tissue** clarification
Step III
Immunotherapy evaluation-CHB
GSE27555
PEG IFN-a treatment responders : 6 samples
Non-responders:7 samples
GSE66698
PEG IFN-a treatment responders
Pre PEG IFN-α treatment :10 samples
Post PEG IFN-α treatment : 7 samples
◆ The role of CLST and aCD4 in evaluating PEG IFN-α sensitivity
Chemotherapy sensitivity prediction-HCC
CHCC
Tumor tissues:159 samples
◆ CLST^high aCD4^high and CLST^low aCD4^low subgroup in response to chemotherapy
Workflow
Stages of HBV infection
1、Microarray
◆ liver tissues
GSE84044
GSE83148
GSE65359
GSE14520
GSE121248
GSE25097
GSE27555
GSE66698
◆ primary human hepatocytes
GSE69590
◆ Human Hepatocyte Chimeric Mice
GSE52752
2、RNA-seq
◆ liver tissues
GSE94660
CHCC
TCGA-LIHC
Our In-house HCC Data

Figure S2

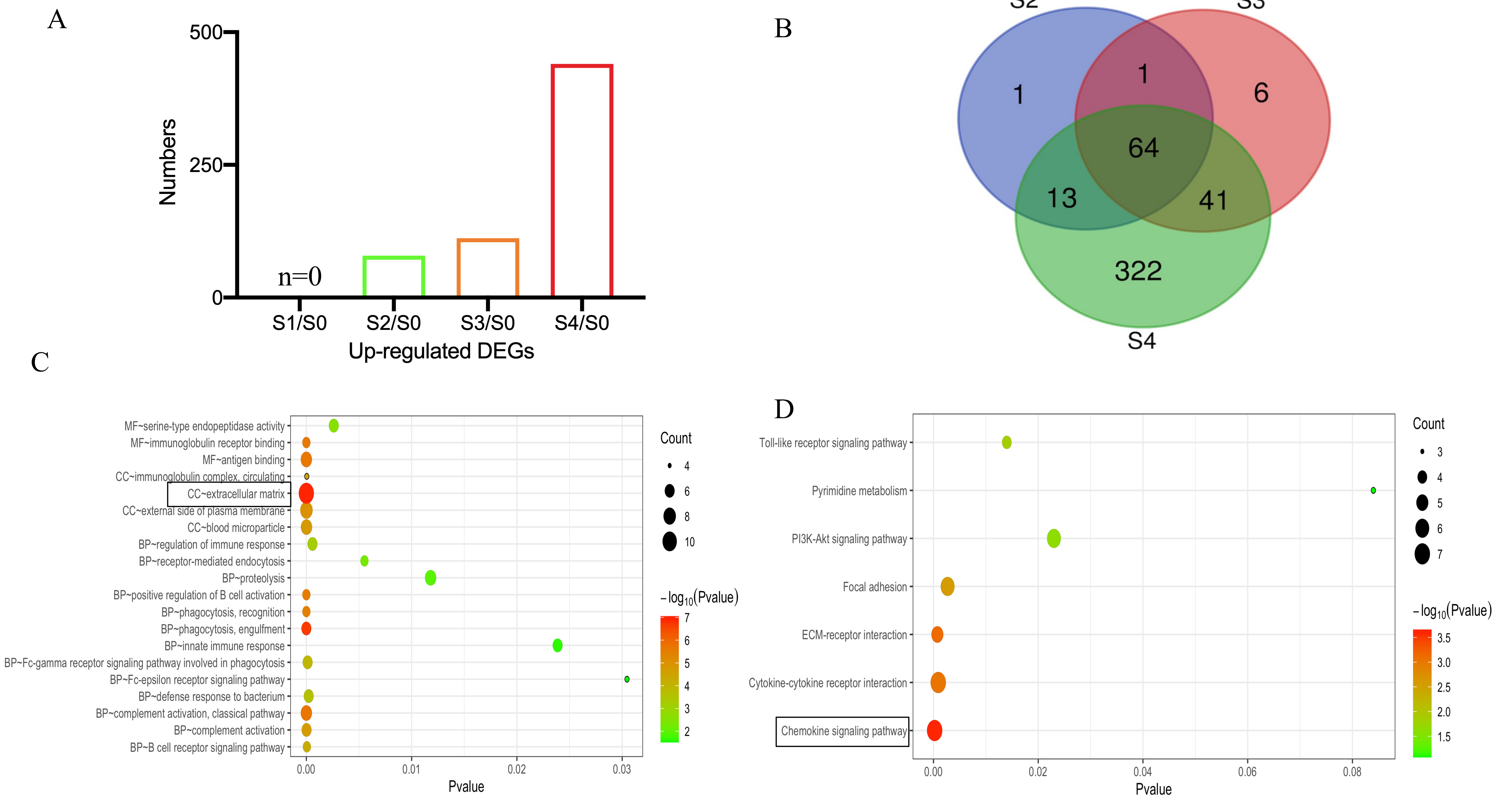
A
500
250
0
Numbers
n=0
S1/S0
S2/S0
S3/S0
S4/S0
Up-regulated DEGs
B
S2
S3
S4
1
1
6
64
13
41
322
C
MF~serine-type endopeptidase activity
MF~immunoglobulin receptor binding
MF~antigen binding
CC~immunoglobulin complex, circulating
CC~extracellular matrix
CC~external side of plasma membrane
CC~blood microparticle
BP~regulation of immune response
BP~receptor-mediated endocytosis
BP~proteolysis
BP~positive regulation of B cell activation
BP~phagocytosis, recognition
BP~phagocytosis, engulfment
BP~innate immune response
BP~Fc-gamma receptor signaling pathway involved in phagocytosis
BP~Fc-epsilon receptor signaling pathway
BP~defense response to bacterium
BP~complement activation, classical pathway
BP~complement activation
BP~B cell receptor signaling pathway
0.00
0.01
0.02
0.03
Pvalue
Count
4
6
8
10
$-\log_{10}$(Pvalue)
7
6
5
4
3
2
D
Toll-like receptor signaling pathway
Pyrimidine metabolism
PI3K-Akt signaling pathway
Focal adhesion
ECM-receptor interaction
Cytokine-cytokine receptor interaction
Chemokine signaling pathway
0.00
0.02
0.04
0.06
0.08
Pvalue
Count
3
4
5
6
7
$-\log_{10}$(Pvalue)
3.5
3.0
2.5
2.0
1.5

Figure S3

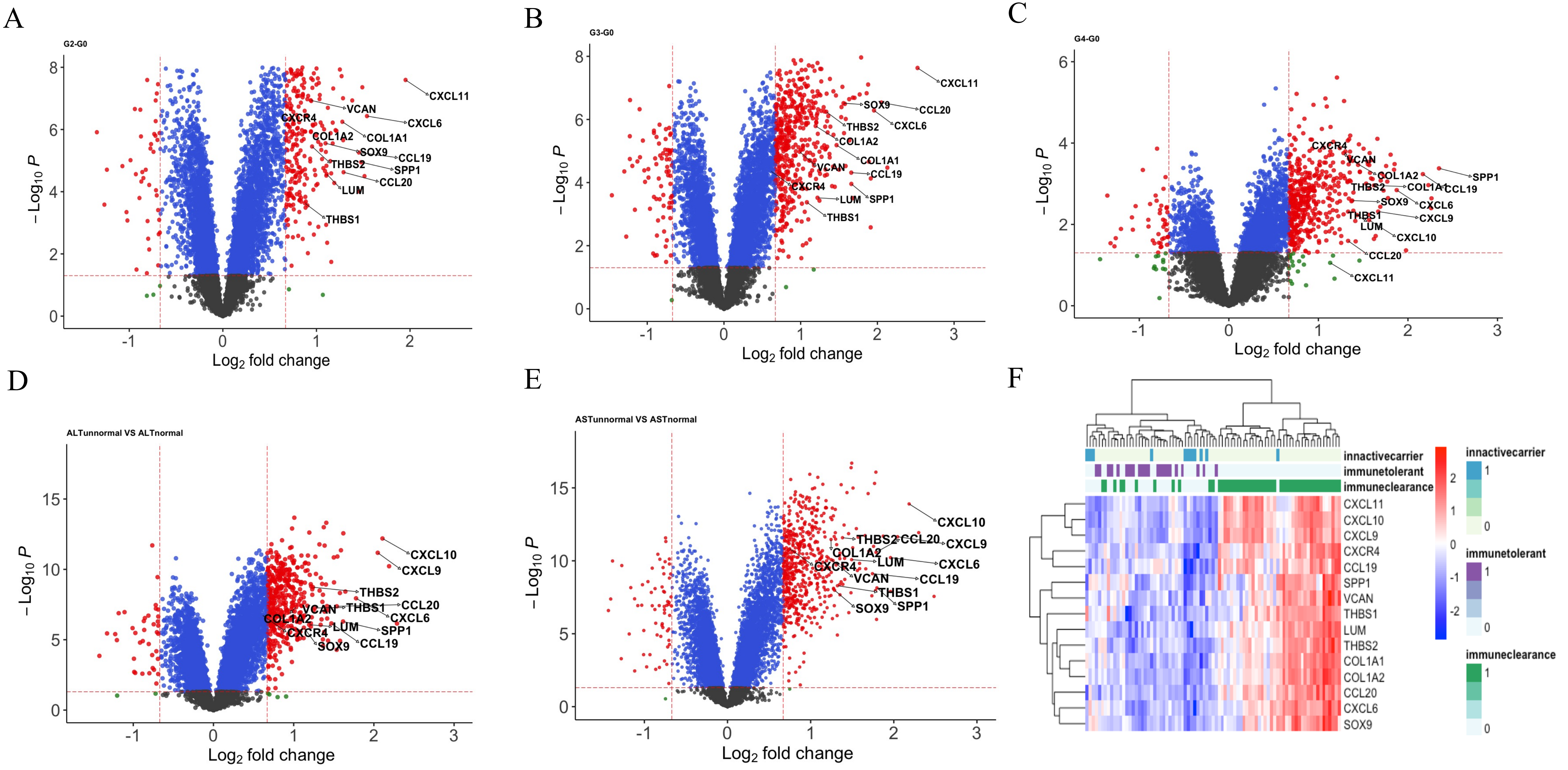
A
G2-G0
CXCL11
VCAN
CXCR4
CXCL6
COL1A1
COL1A2
SOX9
CCL19
THBS2
SPP1
CCL20
LUM
THBS1
$-\log_{10} P$
$\log_2$ fold change
B
G3-G0
CXCL11
SOX9
CCL20
CXCL6
THBS2
COL1A2
COL1A1
VCAN
CCL19
CXCR4
LUM
SPP1
THBS1
C
G4-G0
CXCR4
VCAN
SPP1
COL1A2
THBS2
COL1A1
CCL19
SOX9
CXCL6
THBS1
CXCL9
LUM
CXCL10
CCL20
CXCL11
D
ALTunnormal VS ALTnormal
CXCL10
CXCL9
THBS2
CCL20
VCAN
THBS1
CXCL6
COL1A2
LUM
SPP1
CXCR4
SOX9
CCL19
E
ASTunnormal VS ASTnormal
CXCL10
THBS2
CCL20
CXCL9
COL1A2
LUM
CXCL6
CXCR4
VCAN
CCL19
THBS1
SOX9
SPP1
F
innactivecarrier
immunetolerant
immuneclearance
CXCL11
CXCL10
CXCL9
CXCR4
CCL19
SPP1
VCAN
THBS1
LUM
THBS2
COL1A1
COL1A2
CCL20
CXCL6
SOX9
innactivecarrier
immunetolerant
immuneclearance

Figure S4

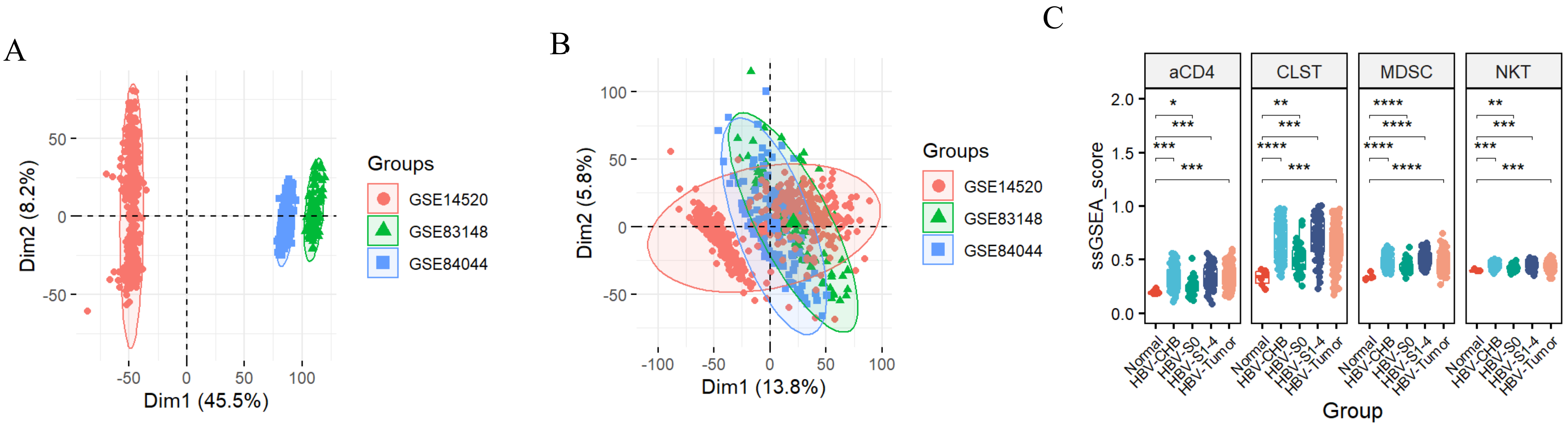
A
Dim2 (8.2%)
Dim1 (45.5%)
Groups
GSE14520
GSE83148
GSE84044
B
Dim2 (5.8%)
Dim1 (13.8%)
Groups
GSE14520
GSE83148
GSE84044
C
aCD4
CLST
MDSC
NKT
ssGSEA_score
Normal
HBV-CHB
HBV-S0
HBV-S1-4
HBV-Tumor
Group

Figure S5

A

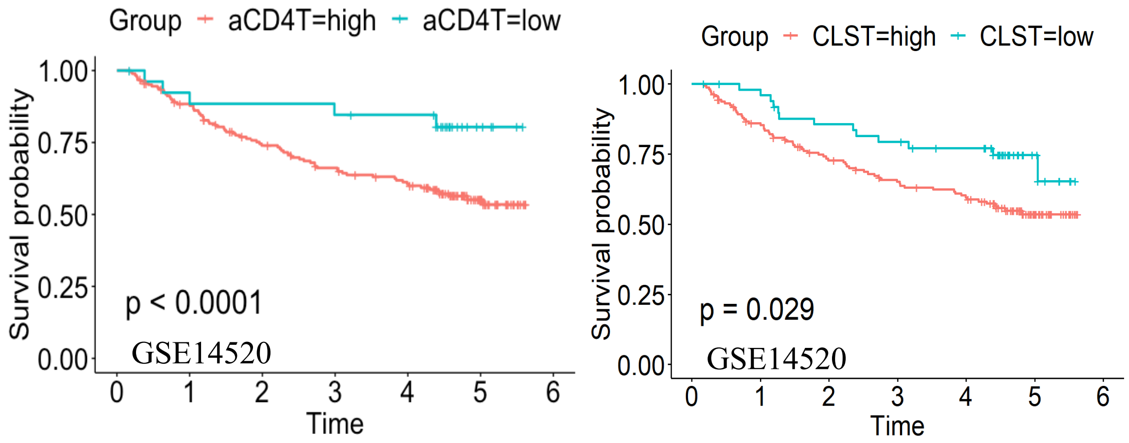
Group aCD4T=high aCD4T=low
Survival probability
1.00
0.75
0.50
0.25
0.00
p < 0.0001
GSE14520
0 1 2 3 4 5 6
Time
Group CLST=high CLST=low
Survival probability
1.00
0.75
0.50
0.25
0.00
p = 0.029
GSE14520
0 1 2 3 4 5 6
Time

B

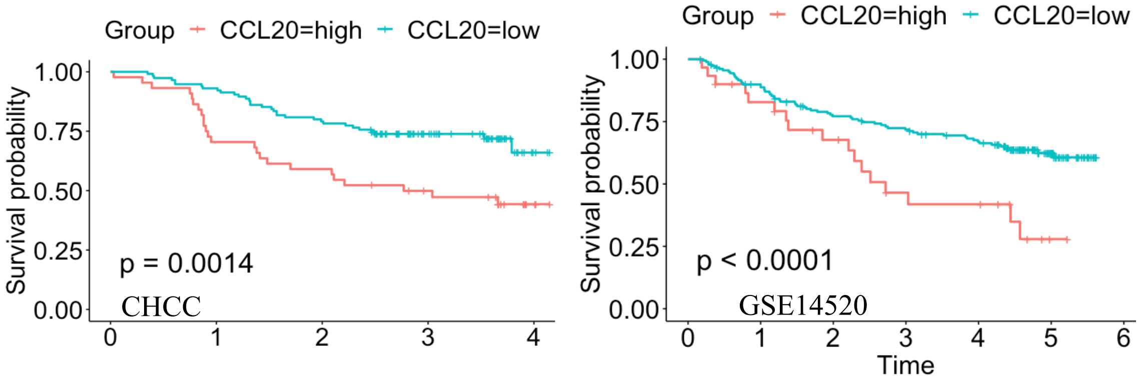
Group CCL20=high CCL20=low
Survival probability
1.00
0.75
0.50
0.25
0.00
p = 0.0014
CHCC
0 1 2 3 4
Group CCL20=high CCL20=low
Survival probability
1.00
0.75
0.50
0.25
0.00
p < 0.0001
GSE14520
0 1 2 3 4 5 6
Time

Figure S6

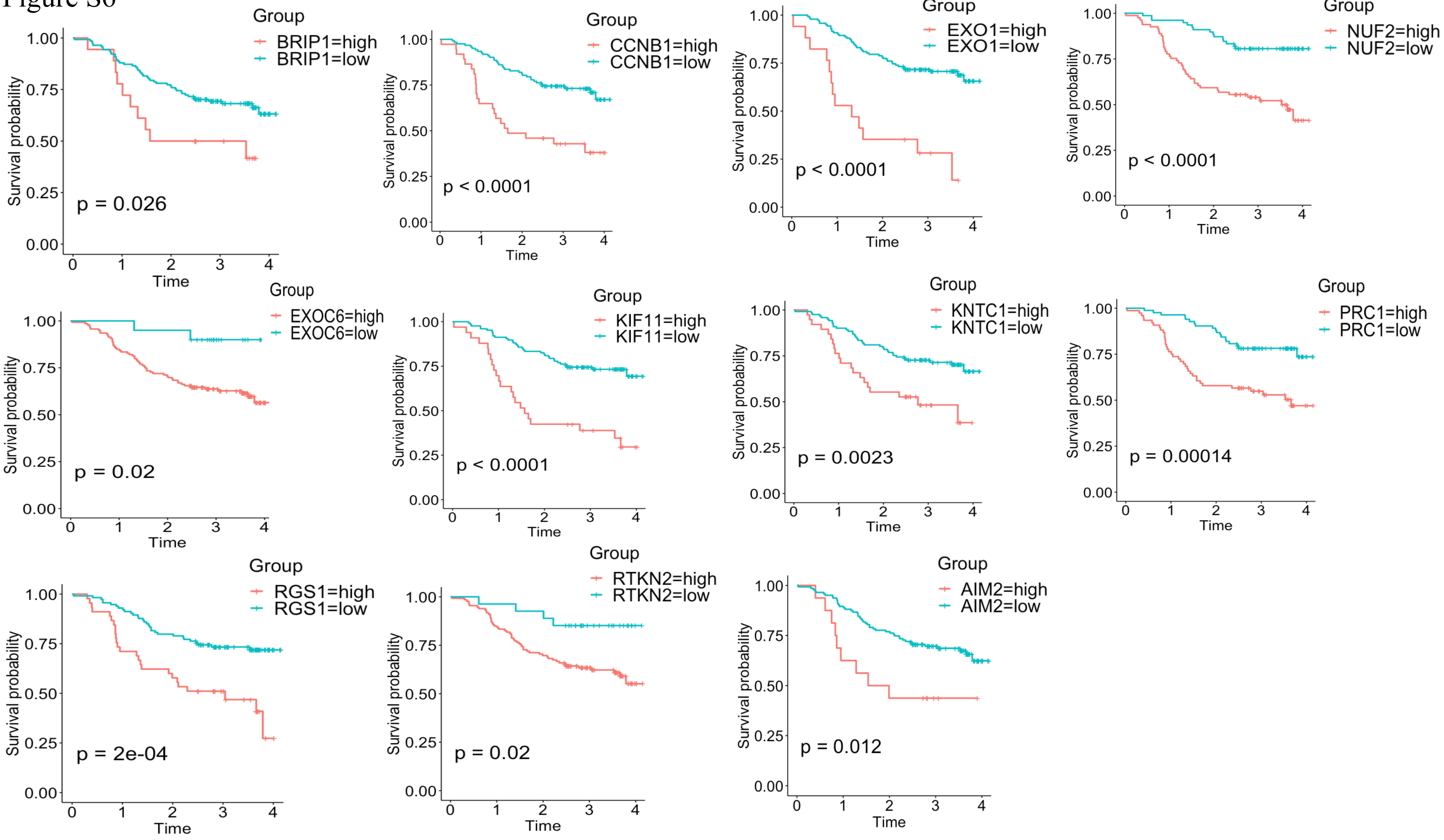
Group
BRIP1=high
BRIP1=low
p = 0.026
Group
CCNB1=high
CCNB1=low
p < 0.0001
Group
EXO1=high
EXO1=low
p < 0.0001
Group
NUF2=high
NUF2=low
p < 0.0001
Group
EXOC6=high
EXOC6=low
p = 0.02
Group
KIF11=high
KIF11=low
p < 0.0001
Group
KNTC1=high
KNTC1=low
p = 0.0023
Group
PRC1=high
PRC1=low
p = 0.00014
Group
RGS1=high
RGS1=low
p = 2e-04
Group
RTKN2=high
RTKN2=low
p = 0.02
Group
AIM2=high
AIM2=low
p = 0.012
Survival probability
Time

Figure S7

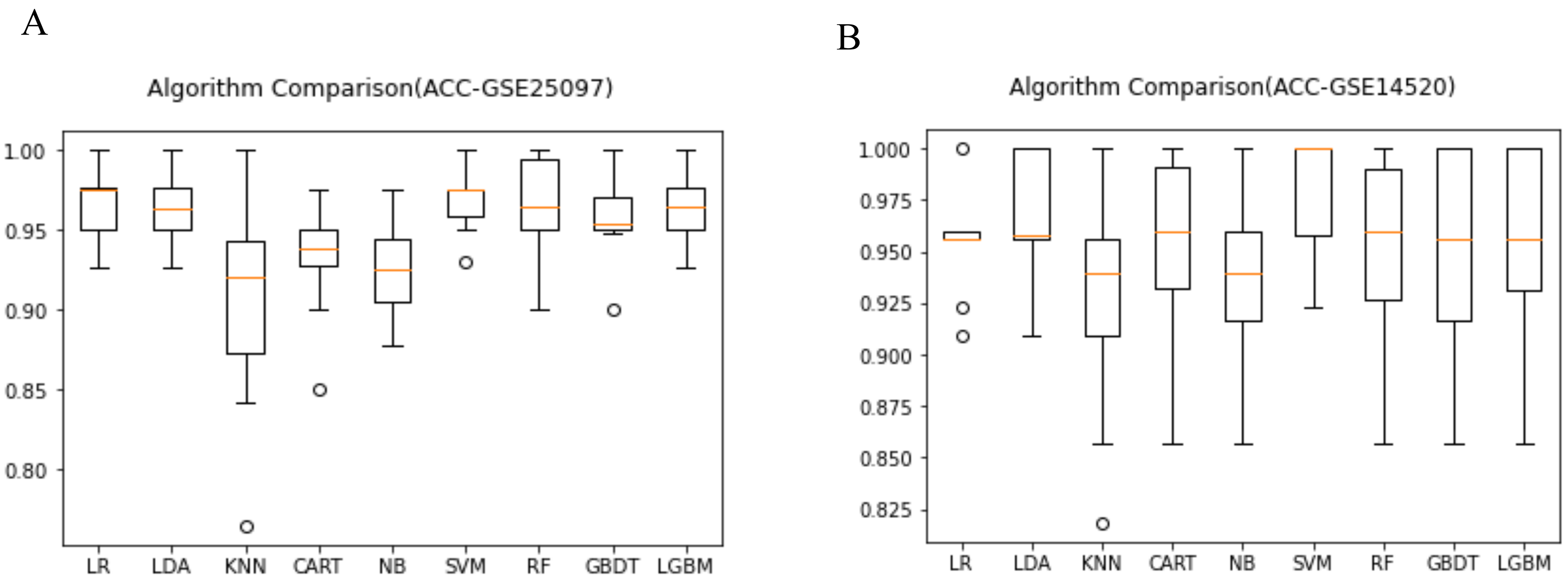
A
Algorithm Comparison(ACC-GSE25097)
1.00
0.95
0.90
0.85
0.80
LR
LDA
KNN
CART
NB
SVM
RF
GBDT
LGBM
B
Algorithm Comparison(ACC-GSE14520)
1.000
0.975
0.950
0.925
0.900
0.875
0.850
0.825
LR
LDA
KNN
CART
NB
SVM
RF
GBDT
LGBM
